# Identification of the Main Chemical constituents and mechanism of Renshen Guben oral liquid against Renal Fibrosis

**DOI:** 10.1186/s13020-023-00762-4

**Published:** 2023-05-17

**Authors:** Junhong Zhang, Juqin Peng, Tong Zhang, Hong Jiang, Yuewen Qin, Hong Chen, Xiaofang Deng, Junguo Ren, Ping Wang, Haiyu Xu

**Affiliations:** 1 Institute of Chinese Materia Medica, China Academy of Chinese Medical Sciences, Beijing, 100700 China; 2 Beijing Key Laboratory of Pharmacology of Traditional Chinese Medicine, Institute of Basic Medical Sciences, Xiyuan Hospital of China Academy of Chinese Medical Sciences, Beijing, 100091 China; 3 Key Laboratory for Research and Evaluation of Traditional Chinese Medicine, National Medical Products Administration, China Academy of Chinese Medical Sciences, Beijing, 100700 China

**Keywords:** RSGB, UPLC-QTOF-MS/MS, UUO model, RNA sequencing, “Constituents-targets-pathways” network

## Abstract

**Background:**

Renal fibrosis is the late stage of many chronic kidney diseases (CKD). Clinically, there is almost no effective treatment for renal fibrosis except dialysis. Renshen Guben oral liquid (RSGB) is a Chinese patent medicine approved by National Medical Products Administration (NMPA), which is suitable for clinical patients with chronic nephritis. Currently, the chemical constituents of RSGB remains unclear, and its efficacy and mechanism on renal fibrosis have not been reported.

**Methods:**

In our research, ultra-high performance liquid chromatography/quadrupole time-of-flight mass spectrometry (UPLC-QTOF-MS/MS) was employed to describe the chemical profile of RSGB, unilateral ureteral obstruction (UUO) model in mice was established to evaluate the beneficial effect of RSGB on renal fibrosis by biochemical indexes, HE and Masson staining. RNA sequencing and “constituents-targets-pathways” multi-dimensional network was established to mine the mechanisms of RSGB. Key targets were verified by quantitative real-time PCR (qRT-PCR) and western bolt (WB).

**Results:**

A total of 201 constituents were identified or tentatively characterized, 15 of which were confirmed with standards. The number of triterpenes was the highest with 49, followed by phenols with 46. RSGB ameliorated the blood urea nitrogen (BUN) and serum creatinine (Scr) levels in serum, normalizing pathological structure of kidney tissue. RNA sequencing revealed that RSGB regulates 226 differential genes, which were involved in kidney development. According to the “constituents-targets-pathways” network, 26 key active constituents may mainly regulate the inflammatory immune system through 88 corresponding targets. qRT-PCR and WB results showed that RSGB inhibited the activation of the Tgfβ1/Smad2/3 pathway, Wnt4/β-Catenin pathway and NGFR/NF-κB pathway.

**Conclusions:**

Overall, our study, for the first time, characterized 201 chemical constituents in RSGB, and 26 of them were screened out to alleviates renal fibrosis mainly through Tgfβ1/Smad2/3 pathway, Wnt4/β-catenin pathway and NGFR/NF-κB pathway, which may provide a new research strategy for research on the mechanism of traditional Chinese Medicine.

**Supplementary Information:**

The online version contains supplementary material available at 10.1186/s13020-023-00762-4.

## Introduction

Renal fibrosis is a common irreversible pathway for the development of chronic kidney disease (CKD) to end stage, characterized by destruction of normal renal structure, fibroblast proliferation, and excessive deposition of extracellular matrix [1, 2]. Clinically, there is almost no effective treatment for renal fibrosis except dialysis. Moreover, available strategies can only ameliorate or delay the progression of CKD, but cannot reverse fibrosis [3]. Unilateral ureteral obstruction (UUO) method has been widely used to establish animal models of interstitial fibrosis. The kidney after ligation usually exhibits several important events, such as inflammation, oxidative stress, macrophage infiltration and fibroblast activation, and activation of the renin-angiotensin-aldosterone system. At the same time, epithelial cells are arrested in the G2/M phase of the cell cycle and adopt a profibrotic secretory phenotype in a variety of kidney injury models. Several molecular mechanisms related to renal interstitial fibrosis have been identified, such as Wnt4, transforming growth factor, beta (TGFβ), Notch, and Hedgehog are critical for renal development, and play positive roles in UUO induced fibrosis models [4, 5].

Renshen Guben oral liquid (RSGB) is a Chinese patent medicine approved by National Medical Products Administration (NMPA), which is suitable for chronic nephritis patients in clinic. RSGB contains 10 herbs, which are respectively from Ginseng radix et rhizoma (*Panax ginseng* C.A.Mey.), Rehmanniae radix (*Rehmannia glutinosa* Libosch), Rehmanniae radix praeparata, Corni fructus (*Cornus officinalis* Sieb. et Zucc.), Dioscoreae rhizoma (*Dioscorea opposita* Thunb.), Moutan cortex (*Paeonia suffruticosa* Andr.), Alismatis rhizoma (*Alisma plantago-aquatica* Linn.), Poria (*Poria cocos* (Schw.) Wolf), Asparagi radix (*Asparagus cochinchinensis* (Lour.) Merr.) and Ophiopogonis radix (*Ophiopogon japonicus* (L. f) Ker-Gawl.). RSGB evolved from Liuwei Dihuang pill (LDP), and the effect of LDP on renal fibrosis has been reported. For example, Shu’s study found that LDP attenuates diabetic nephropathy by inhibiting renal fibrosis through TGF-β/Smad2/3 pathway[6]. Xu et al. demonstrated that LDP prevents renal fibrosis and protects mesangial cells by up-regulating cytoglobin and inhibiting multiple pathways involved in TGF-β/SMADS, MAPK, and NF-κB signaling pathway[7]. At the same time, in the treatment of nephrotic syndrome, the efficacy of RSGB combined with prednisone is significantly better than prednisone alone [8]. Based on this, we suspect that RSGB has potential in the treatment of renal fibrosis.

Currently, RSGB quality standard has been established, which is based on qualitative identification of Ginseng radix et rhizoma and Moutan cortex and quantitative determination of paeonol by UV spectrophotometer. Pharmacological studies have shown that RSGB has obvious anti-fatigue and anti-hypoxia activity [9, 10], and it can also be used as an effective drug for bone marrow suppression in radiotherapy and chemotherapy [11]. However, the chemical constituents of RSGB remains unclear, and its efficacy and molecular mechanism on renal fibrosis have not been reported. Therefore, our objective is to analysis the chemical constituents of RSGB and elucidate its efficacy and mechanism on renal fibrosis.

## Materials and methods

### Chemicals and reagents

RSGB was sponsored by Lunan Hope Pharmaceutical Co., Ltd. (China). Benazepril hydrochloride tablet was purchased from Beijing Novartis pharmaceutical co., ltd. The assay kits for serum creatinine (Scr) and blood urea nitrogen (BUN) were purchased from NJJC Bio (Nanjing, China). HPLC-grade acetonitrile and methanol were purchased from Merck KGaA (Darmstadt, Germany), HPLC-grade formic acid from Sigma-Aldrich (St. Louis, MO, United States). Distilled water was purchased from Watsons Water Co., Ltd. (Shenzhen, China).

Ginsenoside Rg1, ginsenoside Rg2, ginsenoside Rc, ginsenoside Ra1, ginsenoside Rb1 and ginsenoside Re were obtained from Chengdu DeSiTe Biological Technology Co.Ltd. (Chengdu, China). Loganic acid was acquired from Shanghai yuanye Bio-Technology Co Ltd. (Shanghai, China). Gallic acid and paeonol were obtained from Beijing BeiteRenkang Biomedicine Technology Co., Ltd. (Beijing, China). Rehmannioside A, rehmannioside D, loganin, cornuside, apiopaeonoside and paeonolide were purchased form TOPSCIENCE Biochemical Technology Co., Ltd. (Shanghai, China).

### Instrumentation and UPLC-QTOF-MS/MS conditions

The conditions of UPLC-QTOF-MS/MS are in accordance with the previous study [12, 13]. Briefly, the analysis was performed on a Waters Acquity UPLC I-Class system (Waters Corp., Milford, United States), coupled with a Waters QTOF-MS/MS Mass System (Manchester, United Kingdom) equipped with electrospray ionization (ESI). Chromatographic separation was performed on a Waters Acquity UPLC HSS T3 column (100 mm × 2.1 mm, 1.8 μm). Mass range, 50-1500 Da; source temperature 100 °C; desolvation temperature, 450 °C; desolvation gas flow, 900 L/h; sampling cone, 40 V; ESI^−^ capillary voltage, 2.5 KV; and ESI^+^ capillary voltage, 0.5 KV. UPLC-QTOF-MS/MS system was controlled by the Masslynx 4.1 platform. The MS^E^ data collected in a continuum mode were processed using the peak detection and alignment algorithms in UNIFI 1.8, which enabled quasi-molecular ion peaks, adduct ions, and fragment ions to be analyzed as a single entity. RSGB (sugar-free) was filtered through a 0.22 μm microporous membrane before aliquots (2 µL) were transferred to autosampler vials for analysis. Fifteen standards were dissolved in methanol, respectively, and mixed in equal. The final concentration of each standard was 100 ng/mL, and 1µL for analysis.

### UUO model and drug treatment

The animal experiment has been approved by the Animal Ethics Committee of Xiyuan Hospital (No. 2021xlc001-2). The UUO model was established in male C57BL/6J mice (8–10 weeks old), which were purchased from Beijing SiPeiFu Laboratory Animal Technology Co., Ltd. Mice were housed in a normal conditioned environment with a light / dark cycle, 50 ± 5% humidity, 22 ± 2 °C and food and water ad libitum, which were allowed to acclimatize for 2 weeks before the experimentation.

Mice were randomly divided into five groups: Sham, UUO, UUO + RSGB (L), UUO + RSGB (H), UUO + Benazepril. Mice were anesthetized with 1.5-2.0% isoflurane (Beijing ZS Dichuang Technology Development Co., Ltd., Beijing, China), and fixed on homeothermic electric blanket (37 ± 0.5 °C) throughout surgery until coming around. The UUO model was established by cutting the ureter between the two ligations points with 4 − 0 silk thread ligation at the renal pelvis and proximal ureter, respectively. The ureters of Sham mice were exposed, but not ligated. After surgery, the mice were treated with penicillin for 3 days to prevent infection. UUO + RSGB and UUO + Benazepril groups were intragastrical administrated with RSGB or Benazepril daily for 14 days. The kidney and serum were harvested for analysis at the 14th day after surgery.

### HE and Masson staining

Paraffin-embedded kidneys were stained by HE and Masson. The steps of HE staining were as follows: the slices were washed in xylene I for 10 min, xylene II for 10 min, anhydrous ethanol I for 5 min, anhydrous ethanol II for 5 min, 80% alcohol for 5 min, and distilled water successively. Then the slices were dyed with hematoxylin for 5 min, washed with water for 5 min, differentiated with hydrochloric acid ethanol for 1 s, returned with ammonia solution for 10 s, and flushed with water for 30 s. The slice was stained in eosin solution for 1–3 min. Then, the slices were washed in 95% alcohol I for 1 min, 95% alcohol II for 1 min, anhydrous ethanol I for 3 min, anhydrous ethanol II for 3 min, xylene I for 3 min, xylene II for 3 min, and seal the slices with neutral gum.

Masson staining was as follow: the slices were dewaxed to water, nucleated with ferric hematoxylin solution for 5 min, washed with water, differentiated by hydrochloric acid alcohol for several seconds, ponceau solution for 5 min, quickly washed with water, differentiated by 1% phosphomolybdic acid for 2–5 min, aniline blue for 1–3 min, rapidly differentiated by acetic acid, anhydrous ethanol I for 5 min, anhydrous ethanol I for 10 min, transparent, and neutral rubber seal. The percentage of collagen fiber area was analyzed by ImageJ software to obtain the fibrosis rate.

### Scr and BUN assay

The levels of Scr and BUN in serum were measured using commercial kits (NJJC Bio, Nanjing, China) according to the manufacturer’s instructions.

### RNA sequencing and data analysis

Total RNA was extracted from kidney tissue using PEXBIO Cell Total RNA Extraction Kit (APExBIO Technology LLC) and purified with RNeasy mini kit (Qiagen, Valencia, CA, USA). The RNA sequencing libraries were generated using the rRNA-depleted RNA by Directional RNA Library Prep Kit for Illumina (NEB, USA). First strand cDNA was synthesized using random hexamer primer and M-MuLV Reverse Transcriptase (RNase H).

Second strand cDNA synthesis was subsequently performed using DNA Polymerase I and RNase H. PCR was performed with Phusion High-Fidelity DNA polymerase, universal PCR primers and Index (X) Primer. The PCR products were purified (AMPure XP system) and library quality was assessed on the Agilent Bioanalyzer 2100 system. The clustering of the index-coded samples was performed on a cBot Cluster Generation System using Novaseq Cluster Kit (Illumina). After cluster generation, the library preparations were sequenced on an Illumina Novaseq platform. The RNA sequencing was performed by Cnkingbio Corp.

### qRT-PCR

Total RNA was extracted from kidney tissue using PEXBIO Cell Total RNA Extraction Kit (APExBIO Technology LLC), then first-strand cDNA synthesis using a reverse transcription kit (TIANGEN, Beijing, China). qRT-PCR was carried out by using the TB Green (TAKARA, Japan) and real-time fluorescence quantification detected by LightCycler480 System (Roche, Basel, Switzerland). Expression levels were calculated using the 2^–△△Ct^ method, with the Ct values normalized using *GAPDH* as an internal control. The primers used were listed in Additional file 1: Table S1.

### Western blot

Renal tissues were sufficiently lysed with RIPA buffer supplemented with a protease inhibitor cocktail. Protein samples were loaded onto 8%、10% and 12% SDS-PAGE. The protein was transferred to 0.45 μm PVDF membrane, and blocked with 5% skim milk, the primary antibodies against Tgfβ1 (1:1500, ABclonal, A2124), Wnt4 (1:500, Santa Cruz, sc-376,279), Smad2/3 (1:500, Santa Cruz, sc-133,098), NF-κB p-p50 (1:500, Santa Cruz, sc-271,908), β-Catenin (1:10000, Proteintech, 51067-2-AP), DDIT3 (1:3000, Proteintech, 66741-1 g), NGFR (1:1500, Proteintech, 55014-1-AP), NF-κB p65 (1:1500, ABclonal, A18210), NF-κB p-p65 (1:1000, Abcam, ab76302) and β-Tubulin (1:5000, Proteintech, 10068-1-AP) were added and incubated at 4 ℃ overnight. Then, the membranes were probed with HRP-linked antibody (1:5000, BOSTER, BA1054) and incubated at room temperature for 1 h.

### Construction and visualization of multi-dimensional networks

The potential targets of the compounds identified in RSGB were predicted using the TCM target prediction and functional analysis module of TCMIP v2.0 (http://www.tcmip.cn/)[12–14]. The prediction accuracy was set to 0.70 (medium and high similarity). In the disease, syndrome and formula PPI network analysis, the disease genes were the differential genes of UUO/Sham, the syndrome genes were the Qi-Yin Deficiency Syndrome provided by SoFDA platform (http://www.tcmip.cn/Syndrome/front/#/), and the formula genes were the differential genes of RSGB/UUO and the targets of RSGB constituents predicted by TCMIP v2.0. The PPI network were constructed through STRING (https://cn.string-db.org/), and core genes were obtained by screening Degree, Closeness and Betweenness values. Then, the KEGG pathways of key targets were performed by DAVID (https://david.ncifcrf.gov/), and RSGB constituents related to these genes were delineated. Finally, the “constituents-targets-pathways” network was visualized by CytoScape V3.

### Statistical analysis

GraphPad Prism software was used for statistical analysis. One-way ANOVA was used for multi-group comparison. *P* < 0.05 was considered significance.

## Results

### High-throughput identification of chemical constituents in RSGB using UPLC-QTOF-MS/MS

**Fig. 1:**
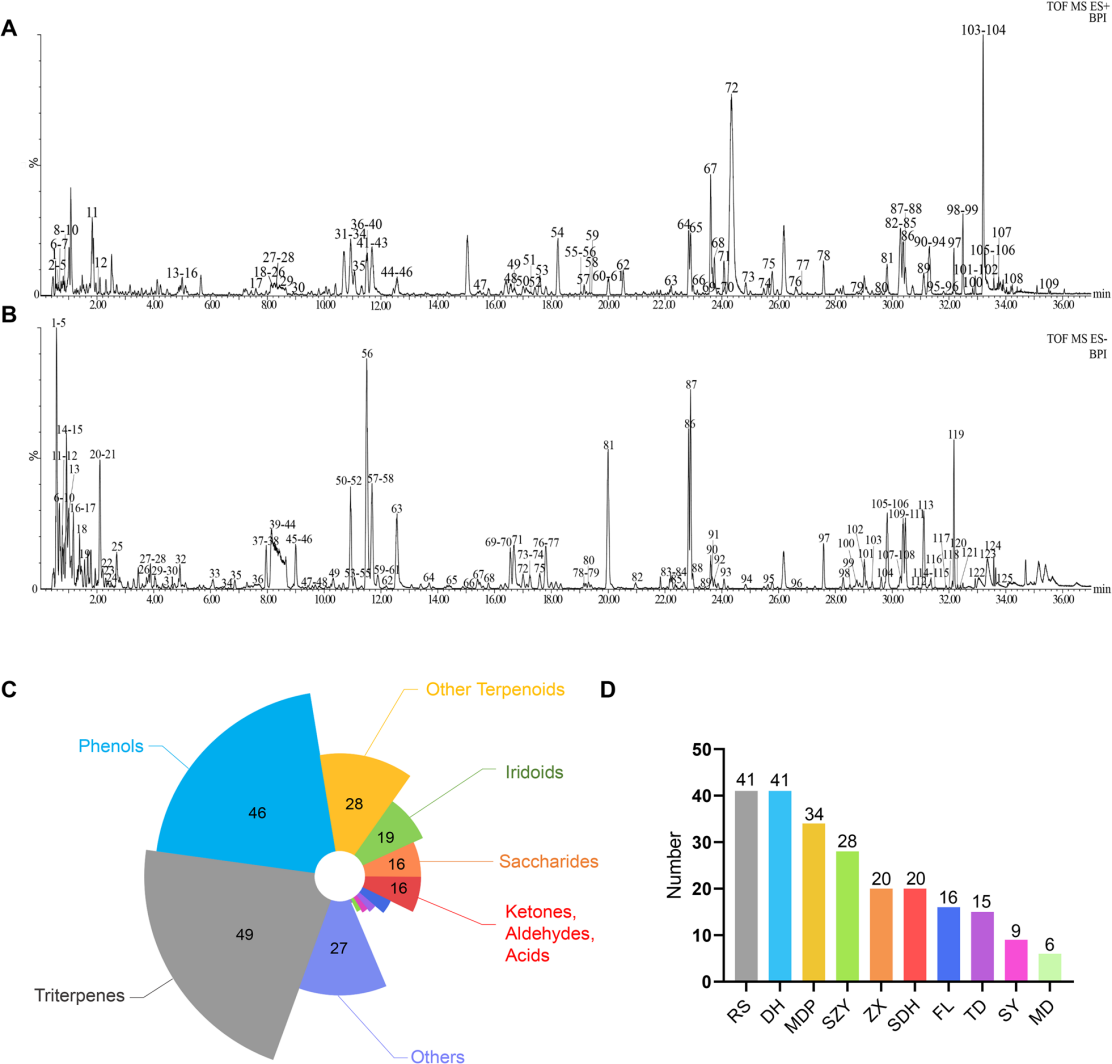
RSGB chemical profile analysis. (**A** and **B**) BPI chromatograms of positive ion mode (**A**) and negative ion mode (**B**); (**C** and **D**) Classification of constituents in RSGB according to chemical structures (**C**) and herbs (**D**). RS is Ginseng radix et rhizoma, DH is Rehmanniae radix, SDH is Rehmanniae radix praeparata, SY is Dioscoreae rhizoma, SZY is Corni fructus, MDP is Moutan cortex, ZX is Alismatis rhizoma, FL is Poria, MD is Ophiopogonis radix, TD is Asparagi radix

In order to describe the chemical fingerprint of RSGB, UPLC-QTOF-MS/MS was used for sample collection and UNIFI 1.8 software for identification [12, 13]. The base peak intensity (BPI) chromatograms of RSGB in positive and negative ion modes were shown in Fig. 1A and B. A total of 201 compounds were identified or tentatively characterized in both the positive (109) and negative (125) ion modes under optimized conditions, including Triterpenes (49), Phenols (46), Other Terpenoids (28), Iridoids (19), Saccharides (16), Ketones, Aldehydes, Acids (16), Phenylpropanoids (9), Organic acids (6), Flavonoids (5), Steroids (4), Sterols (1), Amino acids (1) and Others (27) (Fig. 1C), 41 of which were identified in Ginseng radix et rhizoma, 41 in Rehmanniae radix, 34 in Moutan cortex, 28 in Corni fructus, 20 in Alismatis rhizoma, 20 in Rehmanniae radix praeparata, 16 in Poria, 15 in Asparagi radix, 9 in Dioscoreae rhizome and 6 in Ophiopogonis radix (Fig. 1D). The detailed information of the 201 compounds was listed in Table S2, S3, containing Component name, Observed neutral mass (Da), Observed m/z, Formula, Mass error (ppm), Observed RT (min), Adducts, MS/MS, Category and Herbs.

To further improve the accuracy of the identification, 15 standards were used for comparative analysis, including ginsenoside Rg1, ginsenoside Rg2, ginsenoside Rc, ginsenoside Ra1, ginsenoside Rb1, ginsenoside Re, rehmannioside A, rehmannioside D, loganin, loganic acid, gallic acid, cornuside, paeonol, apiopaeonoside and paeonolide. Altogether 9 compounds in positive ion mode and 13 compounds in negative ion mode corresponded to the standards (Additional file 1: Fig. S1A-1D), thus proving the accuracy of the component identification RSGB.

**Fig. 2:**
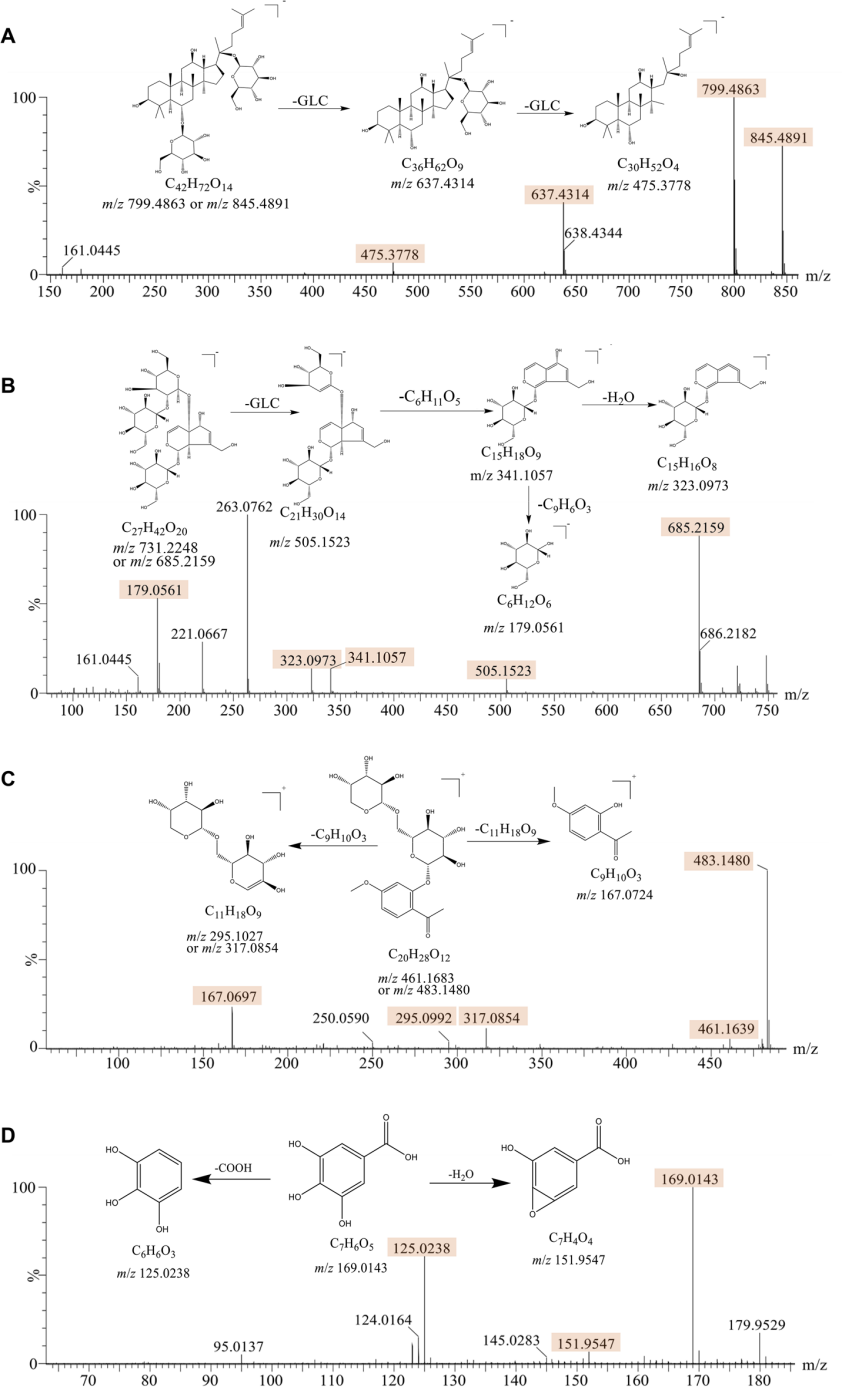
Mass spectrometry of ginsenoside Rg1, rehmannioside D, paeonolide and gallic acid standards and their fragmentation patterns. **A** Ginsenoside Rg1, **B** Rehmannioside D, **C** Paeonolide and **D** gallic acid

Ginsenoside Rg1, rehmannioside D, paeonolide and gallic acid are typical components of Ginseng radix et rhizoma, Rehmanniae radix, Moutan cortex and Corni fructus, respectively, and their fragmentation patterns were analyzed as examples. ⑴ Ginsenoside Rg1 is a triterpene with an accurate molecular weight of 800.4910 (C_42_H_72_O_14_). In the negative ion mode, its quasi-molecular ions were *m*/*z* 799.4863 [M-H]^−^ and *m*/*z* 845.4891[M + HCOO]^−^. As the glycosidic bond breaks, 162-fragments are gradually lost, forming [M-H-Glc]^−^ (C_36_H_62_O_9_, *m*/*z* = 637.4314) and [M-H-2Glc]^−^ (C_30_H_52_O_4_, *m*/*z* = 475.3778) (Fig. 2A). This fragmentation pattern is also applicable to ginsenoside Rb1, Rc, Re, Ra1, etc., so that other triterpenoid saponin can be accurately identified. ⑵ Rehmannioside D is an iridoids with an accurate molecular weight of 686.2260 (C_27_H_42_O_20_). In the negative ion mode, its quasi-molecular ions were [M-H]^−^ (*m*/*z* = 685.2159), and [M + HCOO]^−^ (*m*/*z* = 731.2248). The glycosidic bond of [M-H]^−^ breaks to form [M-H-Glc]^−^ (C_21_H_30_O_14_, *m*/*z* = 505.1523). Then lost a C_6_H_11_O_5_ to form [M-H-2Glc- C_6_H_11_O_5_]^−^ (C_15_H_18_O_9_, *m*/*z* = 341.1057), and lost a C_9_H_8_O_3_ to form [M-H-2Glc-C_6_H_11_O_5_-C_9_H_8_O3]^−^ (C_6_H_12_O_6_, *m*/*z* = 179.0561), or shed a H_2_O to form [M-H-2Glc-C_6_H_11_O_5_-H_2_O]^−^ (C_15_H_16_O_8_, *m*/*z* = 323.0973) (Fig. 2B). According to this pattern, iridoids such as rehmannioside A, catalpol and gardoside could also be identified. ⑶ Paeonolide is a glycoside with an accurate molecular weight of 460.1586 (C_20_H_28_O_12_). In the positive ion mode, paeonolide lost a C_9_H_10_O_3_ to form [M + H-C_9_H_10_O_3_]^+^ (C_11_H_18_O_9_, *m*/*z* = 195.1027), meanwhile, it also can lost a C_11_H_18_O_9_ to form [M + H-C_11_H_18_O_9_]^+^ (C_9_H_10_O_3_, *m*/*z* = 162.0724) (Fig. 2C). Similarly, apiopaeonoside also can be identified. ⑷ Gallic acid is a polyphenol with an accurate molecular weight of 170.1200 (C_7_H_6_O_5_). In the negative ion mode, gallic acid lost a molecule of COOH to form [M-H-COOH]^−^ (C_6_H_6_O_3_, *m*/*z* = 125.0238), or lost a H_2_O to form [M-H-H_2_O]^−^ (C_7_H_6_O_5_, *m*/*z* = 169.0143) (Fig. 2D). Similarly, protocatechuic acid, 4-hydroxybenzoic acid, methyl gallate, etc. can be accurately identified.

### The protective effect of RSGB on mice UUO model

**Fig. 3:**
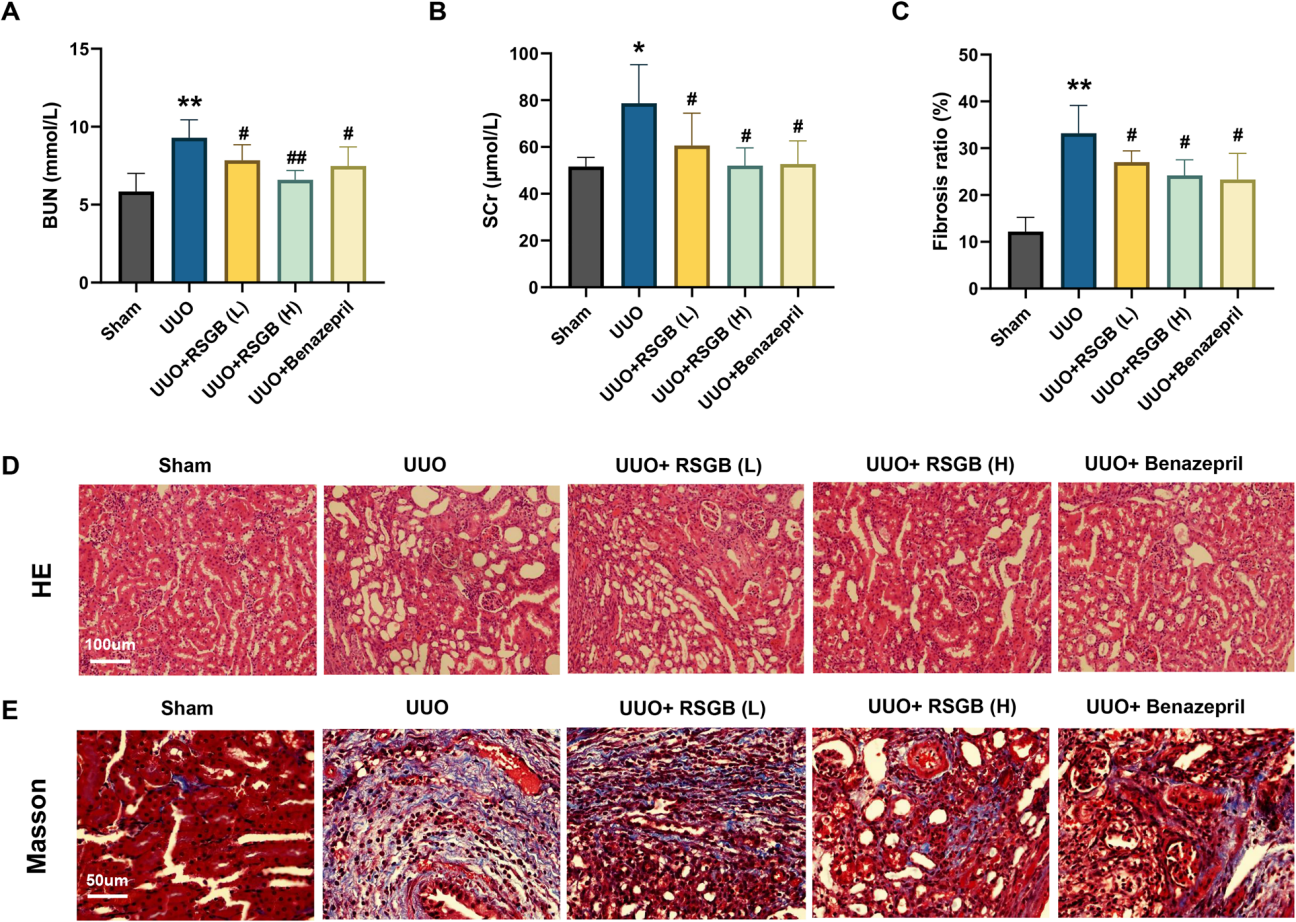
Beneficial effect of RSGB on UUO mice. **A** and **B** The levels of BUN, and Scr in serum detected by biochemical kits; **C** A statistical diagram of fibrosis rate in each group. **D** and **E** HE and Masson staining of renal tissues. **P < 0.01 and *P < 0.05 vs. Sham, ^##^*P* < 0.01 and ^#^*P* < 0.05 vs. UUO.

Ligation of the left ureter is the most common method in UUO model and has been widely adopted to establish animal models of interstitial fibrosis [15]. To investigate the influence of RSGB on renal fibrosis, C57BL/6J mice were treated with RSGB for 14 days after surgery. The results showed that serum Scr and BUN levels of UUO model were significantly increased compared with that of Sham (Fig. 3A and B), which was consistent with the obvious injury in the kidney tissue we have observed. After RSGB treatment, Scr and BUN levels have been significantly suppressed (Fig. 3A and B). HE staining and Masson staining showed that the renal tissue structure of mice in the sham group was normal, no obvious inflammatory cell infiltration was observed in the renal interstitium, and only a small amount of bright blue collagen fibers was observed in the interstitium and around the blood vessels. In the model group, a large number of inflammatory cells were infiltrated into the renal tissue, vacuolar lesions were found in the renal tubules, part of lumen was dilated, the epithelial cells of the renal tubules were atrophied, and there was edema in the renal interstitium, with a large number of blue collagen fibers. However, the above lesions were significantly improved by RSGB and benazepril, the arrangement of tissue cells tended to be orderly, and the bright blue collagen fibers were significantly reduced (Fig. 3C-E). These results indicated that RSGB alleviates renal fibrosis in mice UUO model.

### RNA sequencing analysis of RSGB on renal fibrosis

**Fig. 4:**
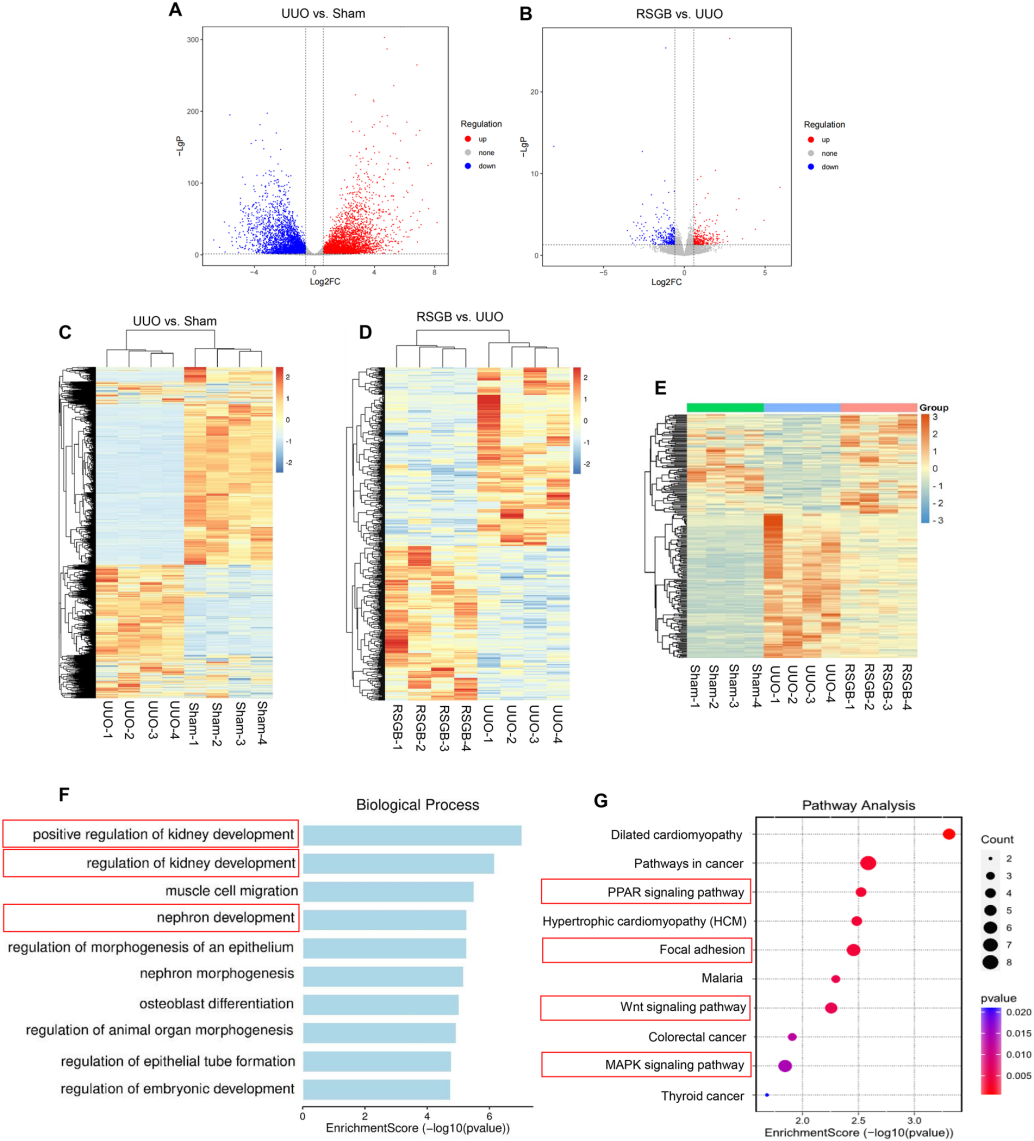
RNA sequencing analysis of RSGB on UUO mice. **A** and **B** Volcano diagram between Sham vs. UUO (**A**) and UUO vs. RSGB (**B**); (**C** and **D**) Heat map between Sham vs. UUO (**C**) and UUO vs. RSGB (**D**); (**E**) Heat map of 226 effect genes in UUO mice after RSGB treatment. (**F**) GO biological process analysis of 226 effect genes of RSGB; (G) KEGG pathway analysis of 226 effect genes

To elucidate the mechanism by which RSGB alleviate renal fibrosis, RNA sequencing was performed (n = 4). As shown in the volcano map and heat map, some mRNA levels in UUO mice altered significantly after RSGB treatment (Fig. 4A-D). Differential gene (FC > 1.5 and *P* < 0.05) screening showed that, a total of 587 genes were regulated by RSGB, among which 226 genes (92 up-regulated and 134 down-regulated) were regulated by RSGB to a level close to that of the Sham group, which were called “direct effector genes” (Fig. 4E). The 226 direct effector genes were subjected to GO analysis, as shown in Fig. 4F, the biological process (BP) included positive regulation of kidney development, regulation of kidney development, and nephron development, indicating the regulation of RSGB on kidney. At the same time, pathway analysis of 226 effector genes was carried out based on KEGG database. The top ten pathways regulated by RSGB include PPAR signaling pathway, Focal adhesion, Wnt signaling pathway and MAPK signaling pathway (Fig. 4G). Importantly, these pathways have been reported to be regulated in kidney disease.

### Multi-dimensional network analysis of RSGB curing renal fibrosis

**Fig. 5:**
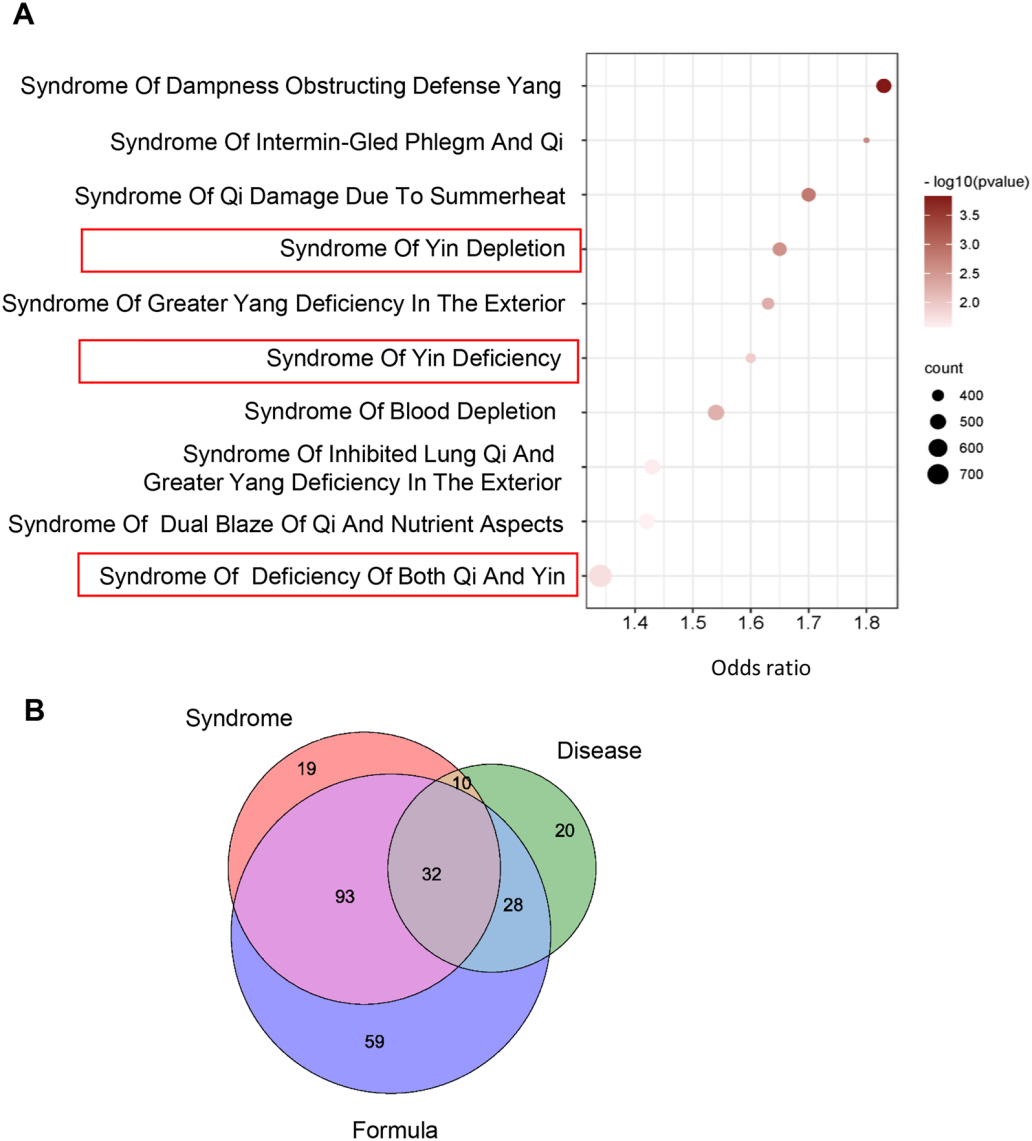
Gene analysis of disease, syndrome and formula. **A** The syndrome enrichment analysis of UUO/Sham differential genes was conducted by SoFDA. **B** KEGG pathway analysis of disease, syndrome and formula genes was carried out by DAVID, and pathways of P < 0.05 were analyzed by Venn diagram

For further elucidate the mechanism of RSGB, Qi-Yin deficiency syndrome (QYDS) (the main syndrome of RSGB treatment) was jointly analyzed. The relationship between UUO model and QYDS was explored by SoFDA (https://cn.string-db.org/). The 743 differential genes (FC > 10) of UUO/Sham were input into SoFDA for TCM syndrome enrichment, and the results showed that they were involved in Syndrome of Yin Depletion (*P* = 0.00277), Syndrome of Yin Deficiency (*P* = 0.012), and Qi-Yin Deficiency Syndrome (*P* = 0.0178) (Fig. 5A), indicating that the UUO model we established conformed to the characteristics of QYDS. Therefore, the combined analysis of disease, syndrome and formula genes were carried out. The genes of three groups were subjected to KEGG pathway analysis, and the pathways with *P* < 0.05 were selected for intersection analysis. As shown in the Venn diagram, disease, syndrome and formula genes had a high correlation (Fig. 5B).

**Fig. 6:**
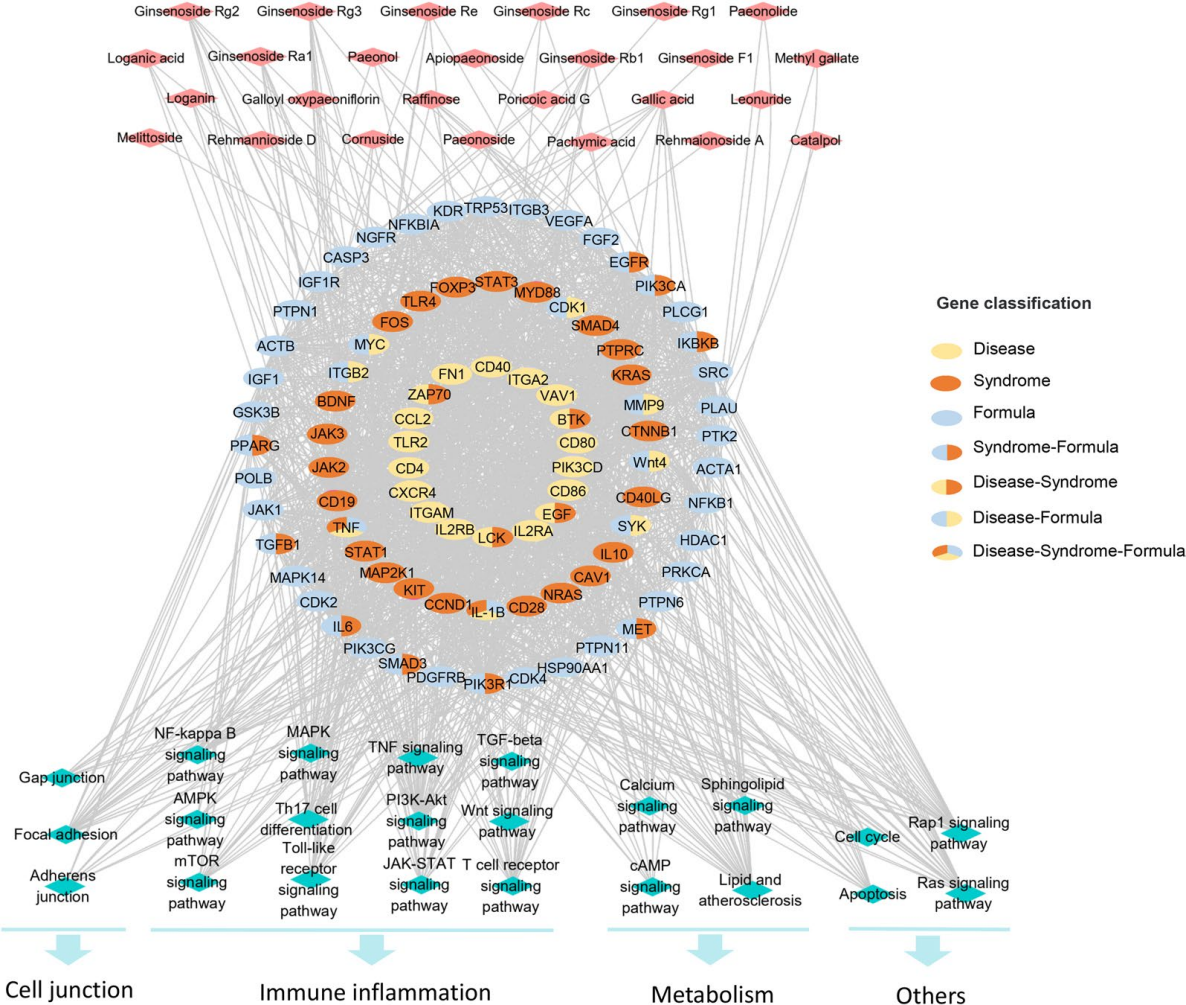
Multi-dimensional network of “Constituents - Targets - Pathways”. Red diamonds represent constituents, ellipses represent targets, and blue diamonds represent pathways. Targets are classified according to their attribution to disease, syndrome, or formula

PPI network analysis of disease, syndrome and formula genes was performed by STRING, and core genes were obtained by screening Degree, Closeness and Betweenness values, and KEGG pathway was enriched by DAVID. Finally, 23 pathways with the highest relevance to UUO model were retained. By analyzing the genes involved in the 23 pathways and RSGB constituents related to these genes, the “constituents-targets-pathways” network was constructed, including 26 key constituents of RSGB and 88 corresponding key genes, which were subdivided 26 disease genes, 27 syndrome genes, and 48 formula genes (Fig. 6). The pathways could be divided into 4 functional modules according to their pharmacological effects: ⑴ Immune and anti-inflammatory related pathways, including NF-kappa B signaling pathway, MAPK signaling pathway, TNF signaling pathway, TGF-beta signaling pathway, AMPK signaling pathway, Th17 cell differentiation, PI3K-Akt signaling pathway, Wnt signaling pathway, mTOR signaling pathway, Toll-like receptor signaling pathway, JAK-STAT signaling pathway and T cell receptor signaling pathway; ⑵ Metabolism related pathway, including Calcium signaling pathway, Sphingolipid signaling pathway, cAMP signaling pathway and Lipid and atherosclerosis; ⑶ Cell junction pathway, including Gap junction, Focal adhesion, Adherens junction; ⑷ Other signaling pathway, including Apoptosis, Cell cycle, Rap1 signaling pathway, Ras signaling pathway.

Among the 26 key constitutes, 8 of them were derived from Ginseng radix et rhizoma (ginsenoside Rg1, ginsenoside Rg2, ginsenoside Rg3, ginsenoside Rb1, ginsenoside Ra1, ginsenoside Rc, ginsenoside Re, ginsenoside F1), 6 from Rehmanniae radix / Rehmanniae radix praeparata (raffinose, leonuride, catalpol, melittoside, rehmannioside D, rehmaionoside A), 5 from Moutan cortex (galloyl oxypaeoniflorin, paeonoside, paeonol, apiopaeonoside, paeonolide), 5 from Corni fructus (cornuside, loganin, loganic acid, gallic acid, methyl gallate), 2 from Poria (poricoic acid G, pachymic acid), 1 from Alismatis rhizoma (raffinose). Some key components of RSGB have been reported to act on anti-inflammatory and renal protection. Ginsenoside (Rg1, Rg2, Rg3, etc.) [16–18], paeonol [19, 20], gallic acid [21], loganin [22] and pachymic acid [23] showed activities in kidney fibrosis and other kidney diseases. Cornuside [24], loganic acid [25] and rehmannioside A [26] have anti-inflammatory activities, which further proved that the key constituents in the “constituents-targets-pathways” network may be used as the pharmacodynamic material basis of RSGB in the treatment of renal fibrosis.

### Mechanism verification of RSGB alleviating renal fibrosis

**Fig. 7:**
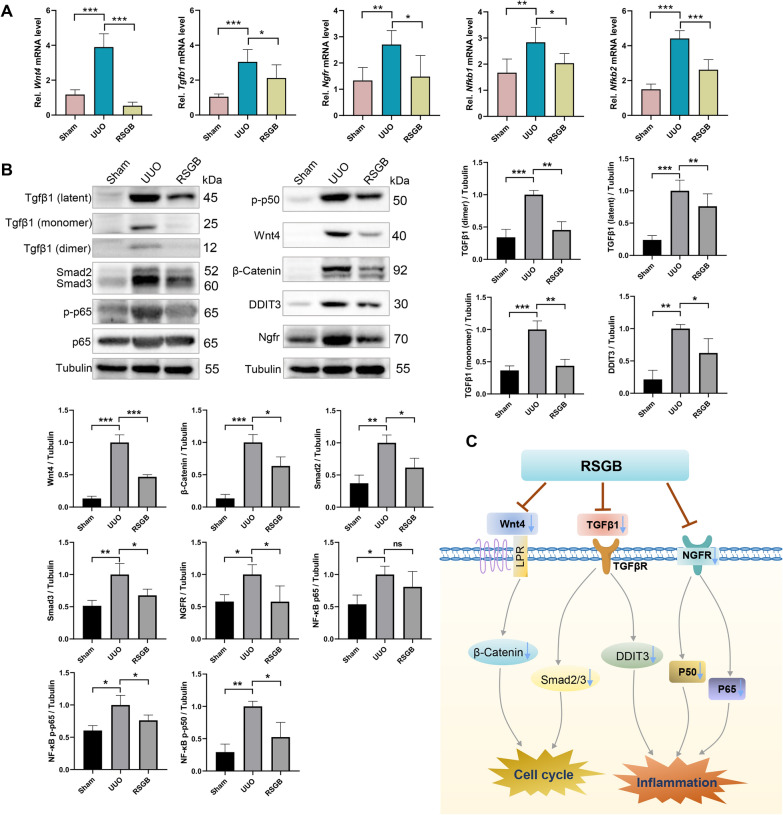
The mechanism of RSGB on UUO model. **A** The genes relative expression levels were detected by qRT-PCR. ^*^P < 0.05, ^**^P < 0.01, ^***^P < 0.001. **B** The levels of Tgfβ1, Smad2/3, Wnt4, β-Catenin, NGFR, NF-κB p65, phospho-NF-kB p65, phospho-NF-kB p50 and DDIT3 in Sham, UUO and RSGB groups were determined by WB. The histograms show the statistics of protein levels (n = 3). ^***^P < 0.001, ^**^P < 0.01, ^*^P < 0.05. **C** The mechanism of RSGB against renal fibrosis

As shown in the “constituents-targets-pathways” multi-dimensional network, RSGB mainly regulates the immune inflammation pathway. Combining differential genes in the RNA sequencing, we selected nuclear factor- kappa B 1 (NFKB1), NFKB2, TGFB1, WNT4, nerve growth factor receptor (NGFR) involved in immune inflammation and cell cycle to perform quantitative real-time PCR (qRT-PCR). The results demonstrated that RSGB significantly altered these gene abnormalities induced by the UUO model (Fig. 7A). In order to further verify the effects of RSGB on TGFβ-1 signaling pathway, Wnt signaling pathway and NF-κB signaling pathway, the expression levels of Tgfβ1, Smad2/3, Wnt4, β-Catenin, NGFR, NF-κB p65, phospho-NF-kB p65, phospho-NF-kB p50 and DDIT3 in Sham, UUO and RSGB groups were determined by WB experiment. In UUO model group, Tgfβ1/Smad2/3 pathway, Wnt4/β-Catenin pathway, and NGFR/NF-κB pathway were activated, which led to inflammatory response and accelerated cell proliferation, thus promoting the progression of renal fibrosis. After treatment with RSGB, the levels of these proteins were significantly reduced (*P* < 0.05), indicating that RSGB inhibited the activation of these pathways (Fig. 7B). Therefore, our study demonstrated that RSGB suppressed the inflammatory response and cell cycle by inhibiting the Tgfβ1/Smad2/3 pathway, Wnt4/β-Catenin pathway and NGFR/NF-κB pathway, thereby exerting an anti-renal fibrosis effect (Fig. 7C).

## Discussion

In this study, we analyzed the chemical constituents of RSGB by UPLC-QTOF-MS/MS, and evaluated the beneficial effect of RSGB on renal fibrosis based on mice UUO model. BUN and Scr levels in serum, and HE and Masson staining of kidney tissue showed RSGB ameliorate renal injury. In order to study the mechanism of RSGB, transcriptome experiment was performed on renal tissues, and construct the “constituents-targets-pathways” multi-dimensional network. Finally, the Tgfβ1/Smad2/3 pathway, Wnt4/β-catenin pathway and NGFR/NF-κB pathway were verified by WB and qRT-PCR.

UPLC-QTOF-MS/MS combined with UNIFI was used for comprehensive and efficient identification of RSGB constituents. The 15 key constituents in RSGB were verified by standards to prove the reliability of identification, and its analogues can be identified by the fragmentation pattern of the standard. In the “constituents-targets-pathways” network, it can be seen that the core components of RSGB in the treatment of renal fibrosis are mainly ginsenoside. Ginsenosides are the main active components of Ginseng radix et rhizoma. Studies have shown that various ginsenosides such as Rb1, Rb3, Rg3, Rh2, Rg1, Rg2 and Rh1 have significant protective effects on renal function, which can reduce renal injury, nephritis, renal fibrosis and renal injury in diabetic nephropathy models [16, 27]. Studies on ginseng showed that Saponins from Panax japonicus ameliorated age-related renal fibrosis by inhibition of inflammation mediated by NF-κB and TGF-β1/Smad signaling and suppression of oxidative stress via activation of Nrf2-ARE signaling [28]. Ginsenoside Rg1 can inhibit the progression of renal fibrosis in the rat model of UUO by regulating the integrity of microvasculature [18], and it can reduce renal injury by inhibiting NOX4-NLRP3 signaling pathway[17]. In the model of acute renal injury, ginsenoside Ro enhanced the anti-inflammatory activity of Achyranthes [29]. In addition to ginsenosides, components of Moutan cortex, Corni fructus, Poria also play an important role. It has been reported that paeonol of Moutan cortex, gallic acid and loganin of Corni fructus are involved in the regulation of NF-κB signaling pathway and other inflammatory pathways [30–32]. Gallic acid has been shown to have a protective effect on kidney during long-term treatment of chronic kidney disease in rats [33]. Paeonol has been proved in UUO model to improve renal interstitial fibrosis by reversing HOTAIR/ Mir-124 /Notch1 axis [20]. Loganin has been reported to alleviate glycolipid toxicity and inflammatory response in liver and kidney of type 2 diabetic mice [34]. Moreover, Pachymic acid of Poria, ameliorated CKD by preventing Wnt/β-catenin activation and RAS gene expression [23]. Meanwhile, maslinic acid and pachymic acid can attenuate acute kidney injury, with maslinic acid by inhibiting NF-KB and MAPK signaling pathways and pachymic acid by inhibiting ferroptosis [35, 36]. Therefore, our analysis of the core constituents of RSGB against renal fibrosis is reliable.

In the UUO model, we demonstrated for the first time that RSGB has the activity of inhibiting renal fibrosis, and analyzed the mechanism of RSGB by RNA sequencing and network analysis. The biological process of GO directly shown the positive regulation of RSGB on the kidney, and the “constituents-targets-pathways” multi-dimensional network indicated that RSGB mainly regulated immune inflammation pathways, such as NF-κB signaling pathway, TGF-β signaling pathway, Wnt signaling pathway, MAPK signaling pathway and PPAR signaling pathway. The NF-κB pathway has been considered as a typical pro-inflammatory signaling pathway, mainly based on the role of NF-κB in the expression of pro-inflammatory genes, including cytokines, chemokines and adhesion molecules [37]. TGF-β is the primary factor that drives fibrosis in most forms of CKD. Inhibition of the TGF-β isoform, TGF-β1, or its downstream signaling pathways substantially limits renal fibrosis [38]. Wnts constitute a family of signaling proteins that play a critical role in kidney development, and Wnt4 has been shown to be reactivated in chronic renal fibrosis [39]. It has been reported that salidroside ameliorates renal interstitial fibrosis by inhibiting the TLR4/NF-κB and MAPK signaling pathways, mindin deficiency alleviates renal fibrosis through inhibiting NF-κB and TGF-β/Smad pathways [40], kallistatin protects renal fibrosis via modulation of Wnt/β-catenin signaling [41], and Zhen-wu-tang ameliorates adenine-induced chronic renal failure in rats by regulating of the canonical Wnt4/beta-catenin signaling in the kidney [42]. Therefore, all of these signaling pathways are potential target pathways for renal fibrosis. In our study, the activation of these pathways was inhibited after RSGB treatment, indicating the excellent efficacy of RSGB.

In conclusion, our study, for the first time, identified the chemical constituents in RSGB by UPLC-QTOF-MS/MS and demonstrate that the improvement of renal fibrosis by RSGB is related to the inhibition of Tgfβ1/Smad2/3 pathway, Wnt4/β-catenin pathway and NGFR/NF-κB pathway.

## Supplementary Information

**Additional file 1: Table S1.** The information of primer sequences. **Table S2.** Identification of chemical constituents of RSGB in positive ion mode. **Table S3. **Identification of chemical constituents of RSGB in negative ion mode. **Figure S1.** Fifteen standard mixtures and RSGB sample were detected by UPLC-QTOF-MS/MS. (A and B) BPI chromatogramsof 15 standard mixtures and RSGB detected in positive ion mode, respectively. (C and D) BPI chromatograms of 15 standard mixtures and RSGB detected innegative ion mode, respectively.

## Data Availability

The data used to support the current study are available from the corresponding author on reasonable request.

## Abbreviations

BUN: Blood urea nitrogen
CKD: Chronic kidney diseases
LDP: Liuwei Dihuang pill
NFKB: Nuclear Factor- Kappa B
NGFR: Nerve growth factor receptor
qRT-PCR: Quantitative real-time PCR
RSGB: Renshen Guben oral liquid
Scr: Serum creatinine
TGFB1: Transforming growth factor, beta 1
UPLC-QTOF-MS/MS: Ultra-high performance liquid chromatography/quadrupole time-of-flight mass spectrometry
UUO: Unilateral ureteral obstruction
