# Supplementary material for: Identification of the Main Chemical constituents and mechanism of Renshen Guben oral liquid against Renal Fibrosis
Source: Chin Med. 2023 May 17;18:56. doi: 10.1186/s13020-023-00762-4 (PMC10190030; doi:10.1186/s13020-023-00762-4)
Supplement: Supplementary file 1 — Additional file 1: Table S1. The information of primer sequences. Table S2. Identification of chemical constituents of RSGB in positive ion mode. Table S3. Identification of chemical constituents of RSGB in negative ion mode. Figure S1. Fifteen standard mixtures and RSGB sample were detected by UPLC-QTOF-MS/MS. (A and B) BPI chromatogramsof 15 standard mixtures and RSGB detected in positive ion mode, respectively. (C and D) BPI chromatograms of 15 standard mixtures and RSGB detected innegative ion mode, respectively. [file 13020_2023_762_MOESM1_ESM.docx]

Supplementary Material

**Table S1**. The information of primer sequences

| Primer name | Sequence (5′-3′) |
| --- | --- |
| NF-κB1 Forward Primer | CAAAGACAAAGAGGAAGTGCAA |
| NF-κB1 Reverse Primer | GATGGAATGTAATCCCACCGTA |
| NF-κB2 Forward Primer | CAAGGACATGACTGCTCAATTT |
| NF-κB2 Reverse Primer | GCCTCTGAAGTTTCTGGATCAT |
| Tgfb1 Forward Primer | ACCGCAACAACGCCATCTATGAG |
| Tgfb1 Reverse Primer | GGCACTGCTTCCCGAATGTCTG |
| Wnt4 Forward Primer | AGTTCAAGCCACATACAGATGA |
| Wnt4 Reverse Primer | TTTAGATGTCTTGTTGCACGTG |
| Ngfr Forward Primer | CCGATGGATCACAAGGTCTAC |
| Ngfr Reverse Primer | GAGCAATAGACAGGAATGAGGT |

**Tab S2.** Identification of chemical constituents of RSGB in positive ion mode.

| NO | **Component name** | **Observed neutral mass (Da)** | **Observed m/z** | **Formula** | **Mass error (ppm)** | **Observed RT (min)** | **Adducts** | **MS/MS** | **Category** | **Herbs** |
| --- | --- | --- | --- | --- | --- | --- | --- | --- | --- | --- |
| 1 | Manninotriose | 504.1685 | 543.1317 | C_18_H_32_O_16_ | -0.9 | 0.54 | +Na | 527.1586[M+Na]; 437.1170; 365.1029 | Saccharides | Rehmanniae radix; Rehmanniae radix praeparata |
| 2 | Trehalose | 342.1168 | 365.1061 | C_12_H_22_O_11_ | 1.7 | 0.62 | +Na | 325.1145; 317.1428; 163.0631; 116.0727 | Saccharides | Poria |
| 3 | Sucrose | 342.1168 | 365.1061 | C_12_H_22_O_11_ | 1.7 | 0.62 | +Na | 145.0509; 127.0434; 97.0291 | Saccharides | Alismatis rhizoma; Rehmanniae radix praeparata |
| 4 | Melibiose | 342.1168 | 365.1061 | C_12_H_22_O_11_ | 1.7 | 0.62 | +Na | 347.0948 | Saccharides | Rehmanniae radix; Rehmanniae radix praeparata |
| 5 | Maltose | 342.1168 | 365.1061 | C_12_H_22_O_11_ | 1.7 | 0.62 | +Na | 163.0631; 145.0509; 127.0434; 91.0432 | Saccharides | Ginseng radix et rhizoma |
| 6 | Raffinose | 504.1691 | 527.1583 | C_18_H_32_O_16_ | 0.1 | 0.66 | +Na | 343.1225; 325.1182 | Saccharides | Alismatis rhizoma; Rehmanniae radix; Rehmanniae radix praeparata |
| 7 | Dextran | 504.1691 | 527.1583 | C_18_H_32_O_16_ | 0.1 | 0.66 | +Na | 343.1225; 325.1182 | Saccharides | Rehmanniae radix |
| 8 | Verbascotetraose | 666.2237 | 689.2129 | C_24_H_42_O_21_ | 2.6 | 0.84 | +Na | 527.1633; 365.1029; 325.1182 | Saccharides | Alismatis rhizoma |
| 9 | Stachyose | 666.2237 | 689.2129 | C_24_H_42_O_21_ | 2.6 | 0.84 | +Na | 527.1633; 365.1029; 325.1182 | Saccharides | Rehmanniae radix; Rehmanniae radix praeparata |
| 10 | Mannan | 666.2237 | 689.2129 | C_24_H_42_O_21_ | 2.6 | 0.84 | +Na | 527.1633; 365.1029; 325.1182 | Saccharides | Dioscoreae rhizoma |
| 11 | Adenosine | 267.0979 | 268.1052 | C_10_H_13_N_5_O_4_ | 4.4 | 1.88 | +H | 136.0639; 85.0308 | Others | Ginseng radix et rhizoma; Moutan cortex; Dioscoreae rhizoma; Rehmanniae radix; Rehmanniae radix praeparata |
| 12 | Guanosine | 283.0927 | 284.1000 | C_10_H_13_N_5_O_5_ | 3.6 | 2.11 | +H | 152.0561; 135.0323; 85.0308 | Others | Rehmanniae radix; Rehmanniae radix praeparata |
| 13 | Santalene | 204.1858 | 227.1750 | C_15_H_24_ | -8.9 | 4.93 | +Na | 183.0333; 153.0165; 97.0291 | Terpenoids | Ginseng radix et rhizoma |
| 14 | α-Humulene | 204.1858 | 227.1750 | C_15_H_24_ | -8.9 | 4.93 | +Na | 183.0305; 153.0165; 97.0291 | Terpenoids | Ginseng radix et rhizoma |
| 15 | Germacrene D | 204.1858 | 227.1750 | C_15_H_24_ | -8.9 | 4.93 | +Na | 183.0333; 153.0165; 97.0291 | Terpenoids | Alismatis rhizoma |
| 16 | 1-Ethenyl 1-Methyl-2,4-Bis(1-Methylethylidene)-Cyclohexane | 204.1858 | 227.1750 | C_15_H_24_ | -8.9 | 4.93 | +Na | 183.0305; 153.0190; 97.0271 | Others | Dioscoreae rhizoma |
| 17 | L-Tyrosine | 181.0731 | 182.0803 | C_9_H_11_NO_3_ | -4.6 | 7.59 | +H | 152.0688; 132.0811; 120.0816 | Amino acid | Asparagi radix; Poria; Rehmanniae radix |
| 18 | Sarracenin | 226.0854 | 227.0927 | C_11_H_14_O_5_ | 5.6 | 8.14 | +H | 195.0679; 139.0421 | Iridoids | Corni fructus |
| 19 | Salicylic Acid | 138.0329 | 139.0402 | C_7_H_6_O_3_ | 8.9 | 8.14 | +H | 121.0680; 97.0311 | [Phenols; Ketones, Aldehydes, Acids](https://www.medchemexpress.cn/NaturalProducts/phenols.html) | Ophiopogonis radix |
| 20 | Protocatechualdehyde | 138.0329 | 139.0402 | C_7_H_6_O_3_ | 8.9 | 8.14 | +H | 121.0680; 103.0559; 91.0589 | Phenols | Poria |
| 21 | Ferulic Acid | 194.0593 | 195.0666 | C_10_H_10_O_4_ | 7.3 | 8.14 | +H | 177.0589; 149.0608; 139.0397 | [Phenols；Phenylpropanoids](https://www.medchemexpress.cn/NaturalProducts/phenylpropanoids.html) | Rehmanniae radix; Rehmanniae radix praeparata; Alismatis rhizoma |
| 22 | Enalin A | 194.0593 | 195.0666 | C_10_H_10_O_4_ | 7.3 | 8.14 | +H | 177.0589; 149.0608; 139.0397 | Phenols | Rehmanniae radix |
| 23 | Caffeic Acid Methyl Ester | 194.0593 | 195.0666 | C_10_H_10_O_4_ | 7.3 | 8.14 | +H | 177.0589; 149.0608; 139.0397 | [Phenols；Phenylpropanoids](https://www.medchemexpress.cn/NaturalProducts/phenylpropanoids.html) | Corni fructus |
| 24 | Butyl Gallate | 226.0854 | 227.0927 | C_11_H_14_O_5_ | 5.6 | 8.14 | +H | 195.0679; 139.0421 | Phenols | Moutan cortex |
| 25 | 4-Hydroxybenzoic Acid | 138.0329 | 139.0402 | C_7_H_6_O_3_ | 8.9 | 8.14 | +H | 95.0459; 77.0407 | [Phenols; Ketones, Aldehydes, Acids](https://www.medchemexpress.cn/NaturalProducts/ketones-aldehydes-acids.html) | Asparagi radix; Rehmanniae radix |
| 26 | 3,4-Dihydroxybenzaldehyde | 138.0329 | 139.0402 | C_7_H_6_O_3_ | 8.9 | 8.14 | +H | 95.0459; 77.0407 | [Phenols; Ketones, Aldehydes, Acids](https://www.medchemexpress.cn/NaturalProducts/ketones-aldehydes-acids.html) | Dioscoreae rhizoma |
| 27 | N-Trans-Feruloyl-N'-Cis-Feruloyl-Cadaverine | 454.2086 | 477.1978 | C_25_H_30_N_2_O_6_ | -3.8 | 8.33 | +Na | 429.1365; 303.0189; 195.0679 | Others | Alismatis rhizoma |
| 28 | Deacyl Martynoside | 476.1905 | 477.1978 | C_21_H_32_O_12_ | 2.4 | 8.33 | +H | 429.1365; 303.0189; 161.0599 | Others | Rehmanniae radix |
| 29 | 7α-Hydroxy-Morroniside | 406.1472 | 429.1364 | C_17_H_26_O_11_ | -0.7 | 8.65 | +Na | 361.0596; 303.0118; 177.0562 | Terpenoids | Corni fructus |
| 30 | Oxypaeoniflorin | 496.1580 | 519.1472 | C_23_H_28_O_12_ | -0.2 | 9.01 | +Na | 179.0740; 151.0762 | Terpenoids; Phenols | Moutan cortex |
| 31 | α-Axetylorcino | 166.0622 | 167.0695 | C_9_H_10_O_3_ | -4.6 | 10.93 | +H | 127.0388; 115.0389; 97.0271 | Phenols | Rehmanniae radix |
| 32 | Paeonolide | 460.1586 | 483.1478 | C_20_H_28_O_12_ | 1.1 | 10.93 | +Na | 167.0722 | Ketones, Aldehydes, Acids | Moutan cortex |
| 33 | Sweroside | 358.1257 | 359.1329 | C_16_H_22_O_9_ | -2 | 10.94 | +H, +Na | 197.0822; 167.0722; 127.0388 | Iridoids | Corni fructus |
| 34 | Paeonilactone B | 196.0733 | 197.0806 | C_10_H_12_O_4_ | -1.3 | 10.94 | +H | 167.0696; 127.0388 | Terpenoids | Moutan cortex |
| 35 | Paeonoside | 328.1162 | 351.1054 | C_15_H_20_O_8_ | 1 | 11.07 | +Na | 239.0029; 220.9918; 153.0216 | Saccharides | Moutan cortex |
| 36 | Jiofuraldehyde Dimethyl Acetal | 228.0992 | 229.1065 | C_11_H_16_O_5_ | -2.4 | 11.5 | +H | 211.0943; 179.0685 | Others | Rehmanniae radix |
| 37 | Hydroferulic Acid | 196.0729 | 197.0802 | C_10_H_12_O_4_ | -3.4 | 11.5 | +H | 179.0713; 161.0625; 1151.0762; 137.0585 | Phenols; Ketones, Aldehydes, Acids | Rehmanniae radix |
| 38 | 3-O-Methylpaeonisuffral | 228.0992 | 229.1065 | C_11_H_16_O_5_ | -2.4 | 11.5 | +H | 211.0943; 179.0685 | Others | Moutan cortex |
| 39 | P-Methoxy Cinnamic Acid | 178.0621 | 179.0694 | C_10_H_10_O_3_ | -5 | 11.51 | +H | 151.0762; 109.0654 | Organic acid | Corni fructus |
| 40 | Coniferyl Aldehyde | 178.0621 | 179.0694 | C_10_H_10_O_3_ | -5 | 11.51 | +H | 161.0573; 151.0737; 109.0633 | Phenols; Phenylpropanoids | Rehmanniae radix |
| 41 | Loganin | 390.1526 | 413.1689 | C_17_H_26_O_10_ | -1.6 | 11.59 | +Na | 179.1277 | Iridoids | Corni fructus |
| 42 | Apiopaeonoside | 460.1570 | 483.1462 | C_20_H_28_O_12_ | -2.3 | 11.66 | +Na +H | 167.0696; 153.0190 | Phenols | Moutan cortex |
| 43 | Homogentisic Acid | 166.0625 | 167.0698 | C_8_H_8_O_4_ | -2.8 | 11.68 | +H | 133.0518; 115.0511; 97.0291 | Phenols; Ketones, Aldehydes, Acids | Rehmanniae radix; Rehmanniae radix praeparata |
| 44 | 2-Phenylethyl-O-Β-D-Xylopyranosyl-(1→6)-O-Caffeoyl-Β-D-Glucopyranoside | 416.1666 | 434.2004 | C_19_H_28_O_10_ | -3.9 | 12.53 | +Na | 339.0147; 179.0713; 151.0762 | Others | Rehmanniae radix |
| 45 | 4-Hydroxy-3-Methoxystyrene | 150.0678 | 151.0750 | C_9_H_10_O_2_ | -2.1 | 12.56 | +H | 133.0659; 105.0702 | Phenols | Ginseng radix et rhizoma |
| 46 | 2-Methoxy-4-Vinylphenol | 150.0678 | 151.0750 | C_9_H_10_O_2_ | -2.1 | 12.56 | +H | 133.0636; 105.0702 | Phenols | Corni fructus |
| 47 | Rehmaionoside A | 390.2242 | 413.2134 | C_19_H_34_O_8_ | -2.9 | 15.48 | +Na | 303.0581; 183.1163 | Terpenoids | Rehmanniae radix; Rehmanniae radix praeparata |
| 48 | Suffruticoside B | 612.1684 | 635.1576 | C_27_H_32_O_16_ | -1 | 16.56 | +Na | 469.0915; 391.1174; 153.0165 | Others | Moutan cortex |
| 49 | Ellagic Acid | 302.0057 | 303.0130 | C_14_H_6_O_8_ | -1.8 | 16.7 | +H | 257.0070; 247.0219 | Phenols | Corni fructus |
| 50 | Albiflorin | 480.1623 | 498.1961 | C_23_H_28_O_11_ | -1.7 | 17.01 | +Na | 503.1580[M+Na]; 179.0713; 151.0762; 133.0659; 105.0343 | Terpenoids | Moutan cortex |
| 51 | Galloyl Paeoniflorin | 632.1725 | 650.2063 | C_30_H_32_O_15_ | -2.5 | 17.26 | +Na | 655.1613[M+Na]; 503.1121; 153.0190 | Phenols | Moutan cortex |
| 52 | Frehmaglutin D | 536.2616 | 537.2689 | C_28_H_40_O_10_ | -1 | 17.42 | +H | 396.1882; 220.0954; 120.0793 | Others | Rehmanniae radix |
| 53 | 7-O-Ethylmorroniside | 434.1776 | 457.1668 | C_19_H_30_O_11_ | -2.7 | 17.81 | +Na | 327.0581; 263.0818; 216.9796 | Iridoids | Corni fructus |
| 54 | Jioglutoside B | 518.1996 | 541.1888 | C_23_H_34_O_13_ | -0.7 | 18.18 | +Na | 322.2503; 228.1610; 209.1662 | Others | Rehmanniae radix |
| 55 | Mudanpioside D | 510.1711 | 511.1784 | C_24_H_30_O_12_ | -5.1 | 19.04 | +H | 378.9995; 303.0439; 181.1033 | Terpenoids | Moutan cortex |
| 56 | 6-O-Trans-Caffeoyl Ajugol | 510.1711 | 511.1784 | C_24_H_30_O_12_ | -5.1 | 19.04 | +H | 478.2164; 345.1381 | Terpenoids | Rehmanniae radix |
| 57 | Jionoside B1 | 814.2887 | 832.3225 | C_37_H_50_O_20_ | -1 | 19.16 | +Na | 837.2805[M+Na]; 691.2233; 529.1713; 339.1089; 177.0562 | [Phenylpropanoids; Phenols](https://www.medchemexpress.cn/NaturalProducts/NaturalProducts/phenols.html.html) | Rehmanniae radix |
| 58 | Mudanpioside H | 616.1781 | 634.2120 | C_30_H_32_O_14_ | -1.7 | 19.32 | +Na | 639.1701[M+Na]; 549.1595; 321.0979 | Terpenoids; Phenols | Moutan cortex |
| 59 | Urolignoside | 522.2094 | 545.1987 | C_26_H_34_O_11_ | -1.2 | 19.42 | +Na | 385.0667; 177.0915; 137.0586 | Others | Corni fructus |
| 60 | Cornuside | 542.1630 | 560.1968 | C_24_H_30_O_14_ | -1 | 20.01 | +H, +Na | 543.1724[M+H]; 211.0972; 179.0685; 153.0190; 141.0544 | Iridoids; Phenols | Corni fructus |
| 61 | 2,4-Dihydroxy-3,5,6-Trimethylmethylbenzoate | 210.0874 | 211.0947 | C_11_H_14_O_4_ | -8.6 | 20.01 | +H | 153.0165; 141.0520 | Others | Rehmanniae radix |
| 62 | Campesterol | 400.3713 | 423.3606 | C_28_H_48_O | 1.9 | 21.85 | +Na | 397.1781; 336.1852; 278.1070 | Steroids | Ginseng radix et rhizoma; Dioscoreae rhizoma |
| 63 | Mudanpioside J | 630.1948 | 648.2287 | C_31_H_34_O_14_ | 0 | 22.37 | +Na | 653.1829[M+Na]; 615.2188; 350.1743; 295.0855 | Terpenoids; Phenols | Moutan cortex |
| 64 | Ginsenoside Rg1 | 800.4901 | 823.4793 | C_42_H_72_O_14_ | -2.6 | 22.83 | +Na, | 643.4193; 603.4247; 441.3755; 423.3614; 405.3517 | Triterpenes | Ginseng radix et rhizoma |
| 65 | Ginsenoside Re | 946.5469 | 969.5361 | C_48_H_82_O_18_ | -3.3 | 22.91 | +Na, +H | 875.3917; 7894783; 549.2698; 441.3712; 423.3614; 405.3476 | Triterpenes | Ginseng radix et rhizoma |
| 66 | Mudanpioside C | 600.1820 | 618.2158 | C_30_H_32_O_13_ | -3.7 | 22.99 | +Na | 623.1722[M+Na]; 335.0964; 265.0736; 191.0675; 121.0297 | Terpenoids; Phenols | Moutan cortex |
| 67 | (25S)-5β-Sprostane-3β-Ol-3-O-β-D-Glucopyranoside | 578.3818 | 579.3890 | C_33_H_54_O_8_ | -0.2 | 23.61 | +H | 417.3370; 273.2209; 255.2092 | Others | Asparagi radix |
| 68 | Parillin | 1048.5489 | 1049.5562 | C_51_H_84_O_22_ | 3.3 | 23.66 | +H, +Na | 957.4102; 795.3710; 579.3858; 417.3370 | Triterpenes | Asparagi radix |
| 69 | Martynoside | 652.2376 | 675.2268 | C_31_H_40_O_15_ | 1.3 | 23.87 | +Na, +H | 529.1666; 417.3370; 273.2175 | Phenylpropanoids；Phenols | Rehmanniae radix praeparata |
| 70 | Isomartynoside | 652.2376 | 675.2268 | C_31_H_40_O_15_ | 1.3 | 23.87 | +Na, +H | 529.1713; 255.2125; 177.0562 | Others | Rehmanniae radix |
| 71 | (25R)-26-O-Β-D-Glucopyranosyl-5Β-Furost-20(22)-En-3Β,26- Dioll-3-O-[Β-D-Glucopyranosyl-(1-2)]-Β-D-Glucopyranoside | 902.4884 | 903.4956 | C_45_H_74_O_18_ | 0.9 | 24.08 | +H, +Na | 741.4365; 579.3907; 273.2209 | Others | Asparagi radix |
| 72 | Paeonol | 166.0635 | 167.0707 | C_9_H_10_O_3_ | 2.8 | 24.34 | +H | 149.0608; 121.0657 | Phenols | Moutan cortex |
| 73 | Benzoyl Paeoniflorin | 584.1896 | 602.2234 | C_30_H_32_O_12_ | 0.3 | 24.85 | +Na | 607.1804[M+Na]; 367.1507; 255.2060; 105.0346 | Terpenoids | Moutan cortex |
| 74 | (25R)-26-O-β-D-Glucopyranosyl-Furost-5,20-Diene-3β,26-Diol-3-O-[α-L- Rhamnopyranosyl(1-2)]-[α-L- Rhamnopyranosyl-(1-4)]-β-D-Glucopyranoside | 1030.5339 | 1031.5411 | C_51_H_82_O_21_ | -1 | 25.48 | +H, +Na | 891.4601; 630.2927; 253.1963 | Others | Asparagi radix |
| 75 | Diosgenin-3-Di-Β-O-Glucopyranoside | 738.4184 | 739.4257 | C_39_H_62_O_13_ | -0.8 | 25.62 | +H | 563.3674; 415.3165; 253.1963 | Others | Dioscoreae rhizoma |
| 76 | Shatavarin Iv | 886.4906 | 887.4979 | C_45_H_74_O_17_ | -2.2 | 26.61 | +H | 741.4420; 255.2125 | Triterpenes | Asparagi radix |
| 77 | (25S)-26-O-β-D-Glucopyranosyl-5β-Furost-20(22)-En-3β,15,26-Triol-3-O-[α-L-Rhamnopyranosyl(1-4)]-β-D- Glucopyranoside | 886.4901 | 887.4974 | C_45_H_74_O_17_ | -2.8 | 26.82 | +H | 621.1408; 255.2125 | Others | Asparagi radix |
| 78 | Ginsenoside Rf | 800.4905 | 823.4797 | C_42_H_72_O_14_ | -2 | 27.59 | +Na, +H | 603.4247; 441.3712; 423.3614; 405.3517 | Triterpenes | Ginseng radix et rhizoma |
| 79 | 9,12,13-Trihydroxy-10-Octadecenoic Acid | 330.2393 | 353.2285 | C_18_H_34_O_5_ | -3.7 | 28.76 | +Na | 303.0474; 213.1297; 175.0768; 147.1185 | Organic acid | Moutan cortex; Rehmanniae radix |
| 80 | Ginsenoside Ra2 | 1210.6327 | 1233.6220 | C_58_H_98_O_26_ | -1.5 | 29.61 | +Na | 875.3856; 789.4783; 467.1391 | Triterpenes | Ginseng radix et rhizoma |
| 81 | Ginsenoside Rb1 | 1108.6028 | 1131.5920 | C_54_H_92_O_23_ | -0.1 | 29.81 | +Na | 649.2248; 325.1145; 425.3806; 407.3688; 487.1626 | Triterpenes | Ginseng radix et rhizoma |
| 82 | Alisol J 23-Acetate | 526.3279 | 527.3352 | C_32_H_46_O_6_ | -2.9 | 30.29 | +H | 415.2828; 353.2520; 215.1449 | Triterpenes | Alismatis rhizoma |
| 83 | Alismanol M | 504.3455 | 505.3527 | C_30_H_48_O_6_ | 0.7 | 30.29 | +H, +Na | 487.3433; 469.3353; 451.3228; 397.2805 | Triterpenes | Alismatis rhizoma |
| 84 | Alismanol E | 468.3245 | 469.3318 | C_30_H_44_O_4_ | 1.2 | 30.29 | +H | 415.2870; 353.2482 | Triterpenes | Alismatis rhizoma |
| 85 | 16-Deoxyporicoic Acid B | 468.3245 | 469.3318 | C_30_H_44_O_4_ | 1.2 | 30.29 | +H | 415.2870; 353.2482 | Triterpenes | Poria |
| 86 | Ginsenoside Ro | 956.5019 | 974.5357 | C_48_H_76_O_19_ | 3.9 | 30.38 | +Na | 979.4873[M+Na]; 439.3573; 321.0823; 204.1859 | Triterpenes | Ginseng radix et rhizoma |
| 87 | Ginsenoside Rc | 1078.5975 | 1101.5867 | C_53_H_90_O_22_ | 4.6 | 30.45 | +Na | 873.3949; 789.4783; 749.4927; 467.1391; 335.0964 | Triterpenes | Ginseng radix et rhizoma |
| 88 | Ginsenoside Ra1 | 1210.6419 | 1233.6311 | C_58_H_98_O_26_ | 5.9 | 30.49 | +Na | 1101.5864; 873.3949; 789.4783; 749.4927; 467.1391; 335.0964 | Triterpenes | Ginseng radix et rhizoma |
| 89 | Ginsenoside Rb2 | 1078.5948 | 1101.5840 | C_53_H_90_O_22_ | 2.2 | 31.11 | +Na | 875.3978; 789.4783; 335.0964 | Triterpenes | Ginseng radix et rhizoma |
| 90 | Biemnasterol | 428.3307 | 451.3199 | C_28_H_44_O_3_ | 3.6 | 31.3 | +Na | 335.0964 | Sterols | Poria |
| 91 | 25-Methoxyporicoic Acid A | 528.3443 | 529.3516 | C_32_H_48_O_6_ | -1.5 | 31.3 | +H | 335.0927 | Triterpenes | Poria |
| 92 | 16β-Hydroperoxyalisol B 23-Acetate | 546.3557 | 569.3449 | C_32_H_50_O_7_ | 0 | 31.3 | +Na, +H | 437.2667; 381.2736; 145.1002 | Triterpenes | Alismatis rhizoma |
| 93 | (22E)-5α,8α-Epidioxyergosta-6,22-Dien-3β-Ol | 428.3307 | 451.3199 | C_28_H_44_O_3_ | 3.6 | 31.3 | +Na | 335.0927; 215.1449; 133.1013 | Steroids | Dioscoreae rhizoma |
| 94 | Ginsenoside Rb3 | 1078.5959 | 1101.5851 | C_53_H_90_O_22_ | 3.2 | 31.36 | +Na | 875.3978; 789.4783; 407.3688; 335.0964 | Triterpenes | Ginseng radix et rhizoma |
| 95 | 16-Oxo-Alisol A 23-Acetate | 546.3561 | 547.3634 | C_32_H_50_O_7_ | 0.8 | 31.81 | +H, +Na | 529.3549; 511.3475; 487.3433; 469.3309; 451.3228 | Triterpenes | Alismatis rhizoma |
| 96 | 16-Oxo-11-Anhydroalisola24-Acetate | 528.3451 | 529.3524 | C_32_H_48_O_6_ | 0.1 | 31.81 | +H | 381.2776; 215.1449; 173.1328 | Triterpenes | Alismatis rhizoma |
| 97 | Ginsenoside Rd | 946.5535 | 969.5427 | C_48_H_82_O_18_ | 3.5 | 32.18 | +Na | 875.3917; 789.4783; 425.3763; 407.3688 | Triterpenes | Ginseng radix et rhizoma |
| 98 | Alisol Q 23-Acetate | 528.3446 | 529.3518 | C_32_H_48_O_6_ | -1 | 32.49 | +H | 487.3433; 469.3309; 451.3228; 433.3092; 415.2807 | Triterpenes | Alismatis rhizoma |
| 99 | Pachymic Acid | 528.7600 | 529.3549 | C_33_H_52_O_5_ | -0.8 | 32.5 | +H | 487.3433; 469.3309; 451.3228; 415.2870 | Triterpenes | Poria |
| 100 | Dehydroeburicoic Acid | 468.3603 | 469.3309 | C_31_H_48_O_3_ | -1.5 | 32.84 | +H | 451.3228; 413.2671; 359.1841 | Triterpenes | Poria |
| 101 | 16-Hydroxyalisol A | 506.3607 | 529.3549 | C_30_H_50_O_6_ | -1.4 | 32.92 | +Na | 489.3601; 471.3456; 453.3378 | Triterpenes | Alismatis rhizoma |
| 102 | 24-Deacetylalisol O | 470.3387 | 471.3460 | C_30_H_46_O_4_ | -1.9 | 32.93 | +H | 453.3378; 395.2926; 381.2776; 339.2596 | Triterpenes | Alismatis rhizoma |
| 103 | Poricoic Acid G | 486.3337 | 487.3409 | C_30_H_46_O_5_ | -1.8 | 33.27 | +H, +Na | 353.2444; 215.1449; 173.1328 | Triterpenes | Poria |
| 104 | Alisol C | 486.3337 | 487.3409 | C_30_H_46_O_5_ | -1.8 | 33.27 | +H, +Na | 469.3309; 451.3228; 397.2764; 376.2597 | Triterpenes | Alismatis rhizoma |
| 105 | Daedaleanic Acid B | 488.3519 | 511.3411 | C_30_H_48_O_5_ | 3.4 | 33.58 | +Na | 425.3806; 365.1029; 184.0757 | Triterpenes | Poria |
| 106 | 15,16-Dihydroalisol A | 488.3519 | 511.3411 | C_30_H_48_O_5_ | 3.4 | 33.58 | +Na | 365.1068; 184.0757 | Triterpenes | Alismatis rhizoma |
| 107 | Alisol L 23-Acetate | 510.3330 | 511.3403 | C_32_H_46_O_5_ | -3 | 33.82 | +H | 313.2737; 282.2850; 184.0757 | Triterpenes | Alismatis rhizoma |
| 108 | Poricoic Acid A | 498.3370 | 521.3262 | C_31_H_46_O_5_ | 4.7 | 34.19 | +Na | 282.2781; 225.1236; 184.0729 | Triterpenes | Poria |
| 109 | Bis(2-Ethylhexyl)Phthalate | 390.2755 | 391.2828 | C_24_H_38_O_4_ | -3.8 | 35.51 | +H, +Na | 149.0233; 279.1583 | Others | Poria |

**Tab S3.** Identification of chemical constituents of RSGB in negative ion mode.

| NO | **Component name** | **Observed neutral mass (Da)** | **Observed m/z** | **_F_ormula** | **Mass error (ppm)** | **Observed RT (min)** | **Adducts** | **MS/MS** | **Category** | **Herbs** |
| --- | --- | --- | --- | --- | --- | --- | --- | --- | --- | --- |
| 1 | β-Hydroxyacteoside | 640.2063 | 699.2202 | C_29_H_36_O_16_ | 8.5 | 0.55 | -H | 639.1990[M-H]; 601.1407; 539.1384; 383.1183 | Others | Rehmanniae radix |
| 2 | Pentosan | 150.0525 | 195.0507 | C_5_H_10_O_5_ | -1.6 | 0.55 | +HCOO | 131.0817; 113.0227; 101.0233 | Saccharides | Asparagi radix |
| 3 | Fructose | 180.0631 | 179.0559 | C_6_H_12_O_6_ | -1.3 | 0.56 | -H | 161.0450; 113.0227; 71.0135 | Saccharides | Ginseng radix et rhizoma |
| 4 | (±)-Tartaric Acid | 150.0162 | 149.0089 | C_4_H_6_O_6_ | -1.8 | 0.58 | -H | 113.0227; 101.0233; 85.0281 | Ketones, Aldehydes, Acids | Ginseng radix et rhizoma |
| 5 | Melibiose | 342.1162 | 377.0856 | C_12_H_22_O_11_ | -0.1 | 0.59 | -H, +HCOO | 341.1067[M-H]; 221.0631; 191.0541; 179.0548; 101.0233; 85.0281; 71.0135 | Saccharides | Rehmanniae radix; Rehmanniae radix praeparata |
| 6 | Trisaccharide | 488.1747 | 533.1729 | C_18_H_32_O_15_ | 1 | 0.61 | +HCOO | 383.1183; 341.1067; 191.0541 | Saccharides | Asparagi radix |
| 7 | Tetrasaccharide | 650.2268 | 695.2250 | C_24_H_42_O_20_ | -0.2 | 0.61 | +HCOO | 503.1621; 383.1183; 191.0541 | Saccharides | Asparagi radix |
| 8 | Leucosceptoside A | 638.2267 | 683.2249 | C_30_H_38_O_15_ | 8.3 | 0.62 | +HCOO | 503.1621; 383.1183; 191.0570 | Phenylpropanoids; Phenols | Rehmanniae radix |
| 9 | Malic Acid | 134.0220 | 133.0147 | C_4_H_6_O_5_ | 3.3 | 0.69 | -H | 115.0041; 89.0233; 87.0095; 71.0135 | [Ketones, Aldehydes, Acids](https://www.medchemexpress.cn/NaturalProducts/ketones-aldehydes-acids.html) | Ginseng radix et rhizoma; Corni fructus |
| 10 | Fumaric Acid | 116.0117 | 115.0044 | C_4_H_4_O_4_ | 6.4 | 0.69 | -H | 71.0135 | Ketones, Aldehydes, Acids | Ginseng radix et rhizoma |
| 11 | Rhoifolin | 578.1675 | 637.1813 | C_27_H_30_O_14_ | 6.1 | 0.78 | -H | 577.1586[M-H]; 503.1621; 341.1067; 179.0548 | Flavonoids | Moutan cortex |
| 12 | Raffinose | 504.1678 | 549.1660 | C_18_H_32_O_16_ | -2.2 | 0.78 | +HCOO, -H | 503.1621[M-H]; 341.1067; 179.0548 | Saccharides | Alismatis rhizoma ; Rehmanniae radix; Rehmanniae radix praeparata |
| 13 | 2'-Acetylacteoside | 666.2190 | 711.2172 | C_31_H_38_O_16_ | 4.2 | 0.85 | +HCOO, -H | 665.2114; 614.1927; 383.1183; 179.0548 | Phenylpropanoids | Ophiopogonis radix |
| 14 | Citric Acid | 192.0279 | 191.0206 | C_6_H_8_O_7_ | 4.6 | 0.93 | -H | 173.0083; 128.0346; 111.0087 | [Ketones, Aldehydes, Acids](https://www.medchemexpress.cn/NaturalProducts/ketones-aldehydes-acids.html) | Ginseng radix et rhizoma |
| 15 | 3-Methyl-2,5-Furandione | 112.0170 | 111.0097 | C_5_H_4_O_3_ | 8.4 | 0.93 | -H | 89.0233; 71.0152 | Others | Dioscoreae rhizoma |
| 16 | Verbascose | 828.2729 | 873.2711 | C_30_H_52_O_26_ | -2.1 | 1.17 | +HCOO, -H | 545.1715; 383.1183; 179.0548; 101.0257 | Saccharides | Rehmanniae radix praeparata |
| 17 | L-Pyroglutamic Acid | 129.0430 | 188.0569 | C_5_H_7_NO_3_ | 2.2 | 1.22 | -H | 128.0346[M-H]; 101.0254; 89.0253 | [Ketones, Aldehydes, Acids](https://www.medchemexpress.cn/NaturalProducts/ketones-aldehydes-acids.html) | Ophiopogonis radix |
| 18 | Succinic Acid | 118.0900 | 117.0184 | C_4_H_6_O_4_ | -1.1 | 1.31 | -H | 89.0233; 71.0135 | Ketones; Aldehydes; Acids | Ginseng radix et rhizoma |
| 19 | Catalpol | 362.1207 | 407.1189 | C_15_H_22_O_10_ | -1.5 | 1.57 | +HCOO, -H | 383.1183; 341.1067; 179.0548 | Iridoids | Rehmanniae radix; Rehmanniae radix praeparata |
| 20 | Gallic Acid | 170.1200 | 169.0147 | C_7_H_6_O_5_ | -1.1 | 2.10 | -H | 125.0233; 113.0248; 101.0233 | Phenols | Corni fructus |
| 21 | Maltol | 126.0320 | 125.0247 | C_6_H_6_O_3_ | 2.6 | 2.11 | -H | 101.0254; 89.0253; 71.0152 | Ketones, Aldehydes, Acids | Ginseng radix et rhizoma |
| 22 | β-Glucogallin | 332.0742 | 331.0670 | C_13_H_16_O_10_ | -0.3 | 2.20 | -H | 271.0467; 169.0147 | Phenols | Moutan cortex |
| 23 | 10-Hydroxyhastatoside | 420.1271 | 465.1253 | C_17_H_24_O_12_ | 0.6 | 2.41 | +HCOO | 341.1105; 179.0548 | Terpenoids | Corni fructus |
| 24 | 7-O-Galloyl-Sedoheptulose | 362.0849 | 361.0776 | C_14_H_18_O_11_ | -0.1 | 2.70 | -H | 271.0433; 211.0232; 179.0548 | Phenols | Corni fructus |
| 25 | 3-O-Galloyl-β-D-Glucose | 332.0743 | 331.0670 | C_13_H_16_O_10_ | -0.1 | 2.80 | -H | 271.0433; 179.0548; 161.0450 | Phenols | Corni fructus |
| 26 | Mudanoside B | 464.1171 | 463.1098 | C_18_H_24_O_14_ | 1.1 | 3.75 | -H | 403.0891; 373.0653; 343.0653; 301.0578; 241.0343; 169.0147 | Terpenoids | Moutan cortex |
| 27 | Rehmannioside D | 686.2260 | 731.2242 | C_27_H_42_O_20_ | -1.3 | 3.88 | +HCOO, -H | 685.2125; 493.1185; 323.0980; 179.0548 | Iridoids | Rehmanniae radix; Rehmanniae radix praeparata |
| 28 | Protocatechuic Acid | 154.1200 | 153.0194 | C_7_H_6_O_4_ | -1.1 | 3.92 | -H | 125.0233; 101.0254 | Phenols; Ketones, Aldehydes, Acids | Moutan cortex |
| 29 | Rehmannioside A | 524.1745 | 569.1727 | C_21_H_32_O_15_ | 0.7 | 4.06 | +HCOO, -H | 523.1689; 493.1276; 373.0817; 169.0120 | Iridoids | Rehmanniae radix; Rehmanniae radix praeparata |
| 30 | Melittoside | 524.1745 | 569.1727 | C_21_H_32_O_15_ | 0.7 | 4.06 | +HCOO, -H | 523.1689; 493.1276; 373.0817; 169.0120 | Iridoids | Rehmanniae radix; Rehmanniae radix praeparata |
| 31 | Mudanoside A | 330.0950 | 329.0877 | C_14_H_18_O_9_ | -0.3 | 4.51 | -H, +HCOO | 221.0661; 169.0120; 108.0207 | Terpenoids; Phenols | Moutan cortex |
| 32 | Leonuride | 348.1421 | 393.1403 | C_15_H_24_O_9_ | 0.3 | 4.88 | +HCOO, -H | 343.0653; 301.0578; 241.0375; 169.0147; 125.0256 | Iridoids | Rehmanniae radix; Rehmanniae radix praeparata |
| 33 | p-Hydroxybenzoic Acid | 138.0318 | 137.0245 | C_7_H_6_O_3_ | 0.8 | 6.08 | -H, +HCOO | 119.0322; 93.0352 | Phenols; Organic acid | Moutan cortex |
| 34 | Aucubin | 346.3300 | 345.1171 | C_15_H_22_O_9_ | -1.4 | 6.63 | -H | 299.1134; 239.0541; 179.0575; 113.0248 | Iridoids | Rehmanniae radix; Rehmanniae radix praeparata |
| 35 | Mussaenosidic Acid | 376.1370 | 375.1297 | C_16_H_24_O_10_ | 0 | 6.86 | -H, +HCOO | 213.0574; 169.0147; 125.0233 | Terpenoids | Rehmanniae radix |
| 36 | Mudanpioside A | 614.2044 | 613.1971 | C_31_H_34_O_13_ | 7.2 | 7.45 | -H | 567.1930; 483.0861; 327.0717 | Terpenoids | Moutan cortex |
| 37 | Loganic Acid | 376.1366 | 375.1294 | C_16_H_24_O_10_ | -0.8 | 7.96 | -H, +HCOO | 213.0753; 169.0866; 151.0772; 113.0248 | Iridoids | Corni fructus |
| 38 | Ajugoside | 390.1523 | 435.1505 | C_17_H_26_O_10_ | -0.8 | 7.98 | +HCOO, -H | 213.0753; 169.0866; 151.0722; 113.0248 | Terpenoids | Rehmanniae radix |
| 39 | Caffeic Acid | 180.0424 | 179.0351 | C_9_H_8_O_4_ | 0.6 | 8.13 | -H | 149.0098; 135.0439 | [Phenylpropanoids; Phenols](https://www.medchemexpress.cn/NaturalProducts/phenylpropanoids.html) | Corni fructus |
| 40 | Paeonisuffrone | 198.0888 | 243.0870 | C_10_H_14_O_4_ | -1.8 | 8.15 | +HCOO | 179.0356; 135.0439 | Terpenoids | Moutan cortex |
| 41 | Rehmapicroside | 346.1625 | 391.1607 | C_16_H_26_O_8_ | -0.7 | 8.18 | +HCOO, -H | 311.0406; 179.0329; 155.0353 | Terpenoids | Rehmanniae radix; Rehmanniae radix praeparata |
| 42 | Morroniside | 406.1477 | 451.1459 | C_17_H_26_O_11_ | 0.4 | 8.43 | +HCOO, -H | 192.0166; 175.0302; 147.0384 | Iridoids | Corni fructus |
| 43 | 8-Epiloganic Acid | 376.1370 | 375.1297 | C_16_H_24_O_10_ | 0.1 | 8.53 | -H | 179.0548; 155.0327; 101.0233 | Iridoids | Rehmanniae radix; Rehmanniae radix praeparata |
| 44 | 7Α-Hydroxy-Morroniside | 406.1476 | 451.1458 | C_17_H_26_O_11_ | 0.3 | 8.65 | +HCOO, -H, | 383.1183; 221.0600; 179.0548 | Iridoids | Corni fructus |
| 45 | Oxypaeoniflorin | 496.1572 | 495.1500 | C_23_H_28_O_12_ | -1.7 | 9.00 | -H, +HCOO | 179.0356; 165.0545; 137.0244 | Terpenoids; Phenols | Moutan cortex |
| 46 | Syringic Acid-4-O-Α-L-Rhamnopyranoside | 344.1101 | 389.1083 | C_15_H_20_O_9_ | -1.6 | 9.02 | +HCOO | 177.0568; 137.0244; 93.0352 | Phenols; Organic acid | Rehmanniae radix |
| 47 | Digalloylglucose | 484.4000 | 483.0771 | C_20_H_20_O_14_ | -1.3 | 9.58 | -H | 331.0660; 169.0120; 125.0256 | Phenylpropanoids | Corni fructus |
| 48 | 2,3-Di-O-Galloyl-D-Glucose | 484.0858 | 483.0785 | C_20_H_20_O_14_ | 0.9 | 9.59 | -H | 385.0772; 169.0147; 125.0233 | Phenols | Corni fructus |
| 49 | 4-Hydroxycinnamic Acid | 164.0474 | 163.0401 | C_9_H_8_O_3_ | 0.5 | 10.32 | -H | 125.0233; 119.0501; 87.0095 | Phenols | Ginseng radix et rhizoma |
| 50 | Geniposide | 388.1370 | 433.1352 | C_17_H_24_O_10_ | 0.1 | 10.91 | +HCOO, -H | 293.0829; 165.0545; 150.0317; 122.0365 | Iridoids | Rehmanniae radix; Rehmanniae radix praeparata |
| 51 | Paeonolide | 460.1584 | 505.1566 | C_20_H_28_O_12_ | 0.6 | 10.93 | +HCOO, -H | 459.1472[M-H]; 293.0864; 165.0545 | Ketones, Aldehydes, Acids | Moutan cortex |
| 52 | Sweroside | 358.1264 | 403.1246 | C_16_H_22_O_9_ | 0.1 | 10.94 | +HCOO, -H | 293.0864; 165.0571; 150.0317 | Phenols | Rehmanniae radix praeparata |
| 53 | Swertiamarine | 374.3400 | 373.1134 | C_16_H_22_O_10_ | -1.6 | 11.07 | -H | 211.0232; 125.0233 | Iridoids | Corni fructus |
| 54 | Geniposidic Acid | 374.1216 | 373.1143 | C_16_H_22_O_10_ | 0.7 | 11.07 | -H | 211.0232; 193.0497; | Iridoids | Rehmanniae radix |
| 55 | Gardoside | 374.1216 | 373.1143 | C_16_H_22_O_10_ | 0.7 | 11.07 | -H | 337.1123; 179.0356; 161.0268 | Iridoids | Rehmanniae radix |
| 56 | Loganin | 390.1526 | 435.2194 | C_17_H_26_O_10_ | -1.1 | 11.62 | +HCOO, -H | 273.9817; 227.0940 | Iridoids | Corni fructus |
| 57 | RFpaeonoside | 460.1580 | 505.1562 | C_20_H_28_O_12_ | -0.1 | 11.68 | +HCOO, -H | 293.0864; 165.0545 | Phenols | Moutan cortex |
| 58 | Trimethyl Citrate | 234.0741 | 293.0879 | C_9_H_14_O_7_ | 0.4 | 11.69 | -H | 233.0685[M-H]; 165.0545; 150.0317; 122.0365 | Organic acid | Poria |
| 59 | Rehmapicrogenin | 184.1101 | 183.1029 | C_10_H_16_O_3_ | 1 | 11.89 | -H, +HCOO | 169.0120; 150.0317; 139.1110 | Ketones, Aldehydes, Acids | Rehmanniae radix |
| 60 | Methyl Gallate | 184.1500 | 183.1009 | C_8_H_8_O_5_ | -1.7 | 11.89 | -H | 165.0545; 150.0317; 135.0439 | Phenols | Corni fructus |
| 61 | 2-Pentylfuran | 138.1046 | 183.1029 | C_9_H_14_O | 1 | 11.89 | +HCOO, -H | 101.0254; 79.9574 | Others | Poria |
| 62 | Syringic Acid | 198.0531 | 197.0458 | C_9_H_10_O_5_ | 1.5 | 12.19 | -H | 153.0524; 137.0244 | Phenols | Corni fructus |
| 63 | Mudanpioside E | 526.1681 | 525.1608 | C_24_H_30_O_13_ | -1 | 12.56 | -H | 515.1324; 479.1549; 449.1470 | Terpenoids; Phenols | Moutan cortex |
| 64 | Galloyloxypaeoniflorin | 648.1684 | 647.1611 | C_30_H_32_O_16_ | -1 | 13.70 | -H | 399.0896; 313.0343; 271.0467; 169.0147 | Phenols | Moutan cortex |
| 65 | 9-Epi-Oxypaeonidanin | 510.1743 | 555.1725 | C_24_H_30_O_12_ | 1.1 | 14.88 | +HCOO | 497.1658; 314.0353 | Terpenoids; Phenols | Moutan cortex |
| 66 | Verbenalin | 388.3700 | 433.1395 | C_17_H_24_O_10_ | -1.1 | 15.21 | +HCOO | 225.0522; 169.0147 | Iridoids | Corni fructus |
| 67 | Rehmaionoside A | 390.2257 | 435.2239 | C_19_H_34_O_8_ | 0.9 | 15.48 | +HCOO, -H | 301.0365; 169.0147 | Terpenoids | Rehmanniae radix |
| 68 | Suffruticoside A | 612.1685 | 611.1612 | C_27_H_32_O_16_ | -0.9 | 15.79 | -H | 445.0974; 300.9974; 169.0545; 165.0545 | Others | Ginseng radix et rhizoma |
| 69 | Suffruticoside B | 612.1703 | 611.1631 | C_27_H_32_O_16_ | 2.1 | 16.56 | -H, +HCOO | 445.1017; 169.0120; 165.0515; | Others | Moutan cortex |
| 70 | Quercetin 3-O-β-D-Glucuronide | 478.0758 | 477.0685 | C_21_H_18_O_13_ | 2.2 | 16.59 | -H | 315.0161; 169.0147; 125.0233 | Flavonoids | Corni fructus |
| 71 | Ellagic Acid | 302.0066 | 300.9993 | C_14_H_6_O_8_ | 1.1 | 16.68 | -H | 283.9987; 169.0120; 125.0233 | Phenols | Corni fructus |
| 72 | Albiflorin | 480.1636 | 525.1618 | C_23_H_28_O_11_ | 0.9 | 17.01 | +HCOO, -H | 479.1504; 449.1470; 135.0439; 121.0294 | Terpenoids | Moutan cortex |
| 73 | Galloyl Paeoniflorin | 632.1737 | 631.1664 | C_30_H_32_O_15_ | -0.7 | 17.25 | -H | 509.2221; 479.1190; 399.0937 | Phenols | Moutan cortex |
| 74 | Engeletin | 434.1214 | 479.1196 | C_21_H_22_O_10_ | 0.2 | 17.25 | +HCOO | 399.0937; 313.0560; 271.0467; 169.0120 | Flavonoids; Phenols | Poria |
| 75 | 7-O-Ethylmorroniside | 434.1786 | 479.1769 | C_19_H_30_O_11_ | -0.3 | 17.81 | +HCOO, -H, | 445.0974; 271.0602; 169.0120 | Iridoids | Corni fructus |
| 76 | Nonanedioic Acid | 188.1047 | 187.0974 | C_9_H_16_O_4_ | -1.1 | 18.16 | -H | 169.0147; 125.0233; 96.9599 | Organic acid | Ginseng radix et rhizoma; Dioscoreae rhizoma |
| 77 | Azelaic Acid | 188.1048 | 187.0975 | C_9_H_16_O_4_ | -1.2 | 18.17 | -H | 169.0120; 125.0210; 101.0233 | Ketones, Aldehydes, Acids | Ophiopogonis radix |
| 78 | Jionoside B1 | 814.2881 | 813.2809 | C_37_H_50_O_20_ | -1.7 | 19.15 | -H, +HCOO | 529.1075; 193.0497; 175.0403 | [Phenylpropanoids; Phenols](https://www.medchemexpress.cn/NaturalProducts/NaturalProducts/phenols.html.html) | Rehmanniae radix |
| 79 | 1,2,3,4,6-Penta-O-Galloyl-β-D-Glucose | 940.1160 | 939.1087 | C_41_H_32_O_26_ | -2.3 | 19.21 | -H | 769.0826; 617.0743; 169.0147 | Phenols | Corni fructus |
| 80 | Mudanpioside H | 616.1795 | 615.1722 | C_30_H_32_O_14_ | 0.4 | 19.31 | -H | 457.0910; 355.0659; 179.0329; 137.0244 | Terpenoids; Phenols | Moutan cortex |
| 81 | Cornuside | 542.1630 | 541.1558 | C_24_H_30_O_14_ | -0.9 | 19.99 | -H | 347.0795; 169.0147; 125.0233 | Iridoids; Phenols | Corni fructus |
| 82 | Hesperidin | 610.1886 | 609.1813 | C_28_H_34_O_15_ | -1.9 | 20.96 | -H, +HCOO | 300.9939; 286.0493; | Flavonoids | Poria |
| 83 | Ginsenoside I | 978.5377 | 977.5304 | C_48_H_82_O_20_ | -2.3 | 22.17 | -H | 931.5237; 771.4133; 475.3782; 391.2880 | Triterpenes | Ginseng radix et rhizoma |
| 84 | Bornyl 7-O-α-L-Arabinofuranosyl-(1→6)- β-D-Glucopyranoside | 448.2308 | 493.2277 | C_21_H_36_O_10_ | -1.1 | 22.24 | +HCOO | 447.2260; 281.0688; 137.0244 | Others | Corni fructus |
| 85 | Mudanpioside J | 630.1951 | 629.1878 | C_31_H_34_O_14_ | 0.3 | 22.36 | -H, +HCOO | 507.1535; 493.2277; 461.2387 | Terpenoids; Phenols | Moutan cortex |
| 86 | Ginsenoside Rg1 | 800.4910 | 845.4892 | C_42_H_72_O_14_ | -1.4 | 22.83 | +HCOO, -H | 799.4832; 637.4344; 475.3827; 391.2880 | Triterpenes | Ginseng radix et rhizoma |
| 87 | Ginsenoside Rd | 946.5478 | 991.5460 | C_48_H_82_O_18_ | -2.4 | 22.90 | +HCOO, -H | 783.4924; 637.4344; 475.3782 | Triterpenes | Ginseng radix et rhizoma |
| 88 | Benzoyl Oxypaeoniflorin | 600.1840 | 599.1767 | C_30_H_32_O_13_ | -0.6 | 22.98 | -H,+HCOO | 475.3827; 457.3669; 391.2110; 137.0244 | Terpenoids; Phenols | Moutan cortex |
| 89 | Paeoniflorin B | 746.2403 | 791.2385 | C_36_H_42_O_17_ | -2.3 | 23.42 | +HCOO, -H | 601.3014; 407.1707 | Terpenoids | Moutan cortex |
| 90 | Shatavarin I | 1066.5522 | 1111.5504 | C_51_H_86_O_23_ | -3.4 | 23.66 | +HCOO, -H | 757.4368; 595.3892; 433.3314 | Terpenoids | Asparagi radix |
| 91 | Asparaside A | 1066.5526 | 1111.5508 | C_51_H_86_O_23_ | -3.1 | 23.79 | +HCOO, -H | 919.4973; 757.4368; 595.3892; 433.3271 | Terpenoids | Asparagi radix |
| 92 | Martynoside | 652.2364 | 651.2291 | C_31_H_40_O_15_ | -0.5 | 23.87 | -H, +HCOO | 595.3842; 433.3314; 193.0525 | [Phenylpropanoids, Phenols](https://www.medchemexpress.cn/NaturalProducts/NaturalProducts/phenols.html.html) | Rehmanniae radix praeparata |
| 93 | 26-O-β-D-Glucopyranosyl-Furost-3β,22,26-Triol-3-O-β-D-Glucopyranosyl(1→2)-O-β-D-Glucopyranoside | 920.4962 | 965.4944 | C_45_H_76_O_19_ | -1.9 | 24.08 | +HCOO, -H | 919.4849; 757.4368; 595; 3892; 433.3357 | Others | Asparagi radix |
| 94 | Benzoyl Paeoniflorin | 584.1894 | 629.1876 | C_30_H_32_O_12_ | 0 | 24.84 | +HCOO, -H | 352.5655; 261.0014; 121.0294 | Terpenoids | Moutan cortex |
| 95 | Parisaponin I | 1034.5298 | 1079.5315 | C_50_H_82_O_22_ | -1.5 | 25.64 | +HCOO | 1033.5222; 901.4874; 755.4200; 575.3688 | Steroids | Moutan cortex |
| 96 | (25S)-26-O-β-D-Glucopyranosyl-5β-Furostan-3β,22α,26-Triol-3-O-β-D-Glucopyranoside | 904.4992 | 949.4974 | C_45_H_76_O_18_ | -4.2 | 26.61 | +HCOO, -H | 810.3501; 757.4315; 433.3357 | Others | Asparagi radix |
| 97 | Ginsenoside Rf | 800.4886 | 845.4868 | C_42_H_72_O_14_ | -4.3 | 27.59 | +HCOO, -H | 799.4832; 637.4292; 475.3782; 391.2839 | Triterpenes | Ginseng radix et rhizoma |
| 98 | Ginsenoside F3 | 770.4802 | 815.4785 | C_41_H_70_O_13_ | -1.7 | 28.27 | +HCOO, -H | 637.4447; 475.782 | Triterpenes | Ginseng radix et rhizoma |
| 99 | Ginsenoside Ra3 | 1240.6442 | 1285.6424 | C_59_H_100_O_27_ | -0.8 | 28.51 | +HCOO, -H | 1107.5935; 945.5420; 783.4924; 621.4374 | Triterpenes | Ginseng radix et rhizoma |
| 100 | 9,12,13-Trihydroxy-10-Octadecenoic Acid | 330.2409 | 329.2337 | C_18_H_34_O_5_ | 1 | 28.75 | -H | 240.9962; 178.8411; 121.0317 | Organic acid | Moutan cortex; Rehmanniae radix |
| 101 | Ginsenoside Rg2 | 784.4792 | 829.4931 | C_42_H_72_O_13_ | -2.9 | 29.02 | +HCOO, -H | 783.4752; 637.4292; 475.3827; 391.2839 | Triterpenes | Ginseng radix et rhizoma |
| 102 | Ginsenoside F1 | 638.4378 | 683.4360 | C_36_H_62_O_9_ | -2.3 | 29.10 | +HCOO | 475.3782 | Triterpenes | Ginseng radix et rhizoma |
| 103 | 20(R)Ginsenoside Rg2 | 784.4942 | 829.4924 | C_42_H_72_O_13_ | -3.8 | 29.29 | +HCOO, -H | 783.4867; 475.3782; 391.2839 | Triterpenes | Ginseng radix et rhizoma |
| 104 | Ginsenoside Ra2 | 1210.6313 | 1255.6295 | C_58_H_98_O_26_ | -2.6 | 29.61 | +HCOO, -H | 1077.5807; 945.5420; 783.4924; 621.4374 | Triterpenes | Ginseng radix et rhizoma |
| 105 | Notoginsenoside R4 | 1240.6435 | 1285.6417 | C_59_H_100_O_27_ | -1.3 | 29.76 | +HCOO, -H | 1107.5935; 945.5420; 783.4982; 621.4374 | Triterpenes | Ginseng radix et rhizoma |
| 106 | Ginsenoside Rb1 | 1108.6014 | 1153.5996 | C_54_H_92_O_23_ | -1.3 | 29.82 | +HCOO, -H | 1077.5935; 945.5420; 783.4924; 621.4374; 459.3842 | Triterpenes | Ginseng radix et rhizoma |
| 107 | Malonyl Ginsenoside Rb1 | 1194.6002 | 1193.5929 | C_57_H_94_O_26_ | -2.6 | 30.22 | -H | 1107.6072; 945.5357; 783.4867 | Triterpenes | Ginseng radix et rhizoma |
| 108 | 16-Oxoalisol A | 504.3446 | 549.3428 | C_30_H_48_O_6_ | -0.9 | 30.29 | +HCOO, -H | 455.3541; 295.1695 | Triterpenes | Alismatis rhizoma |
| 109 | Ginsenoside Ro | 956.4957 | 955.4884 | C_48_H_76_O_19_ | -2.5 | 30.38 | -H, +HCOO | 793.4367; 569.3854; 455.3541 | Triterpenes | Ginseng radix et rhizoma |
| 110 | Ginsenoside Rc | 1078.5900 | 1123.5882 | C_53_H_90_O_22_ | -2.1 | 30.46 | +HCOO, -H | 1077.5807; 945.5420; 783.4867; 621.4374; 459.3798 | Triterpenes | Ginseng radix et rhizoma |
| 111 | Ginsenoside Ra1 | 1210.6315 | 1255.6297 | C_58_H_98_O_26_ | -2.5 | 30.49 | +HCOO, -H | 1077.5807; 945.5420; 783.4867; 621.4374 | Triterpenes | Ginseng radix et rhizoma |
| 112 | Malonyl Ginsenoside Rc | 1164.5913 | 1163.5841 | C_56_H_92_O_25_ | -1.2 | 30.88 | -H | 1077.5874; 1059.5630; 9455294; 783.4867; 621.4426; 459.3842 | Triterpenes | Ginseng radix et rhizoma |
| 113 | Ginsenoside Rb2 | 1078.5908 | 1123.5890 | C_53_H_90_O_22_ | -1.4 | 31.11 | +HCOO, -H | 1077.5807; 945.5357; 783.4867; 621.4374 | Triterpenes | Ginseng radix et rhizoma |
| 114 | 16β-Hydroperoxyalisol B 23-Acetate | 546.3549 | 591.3531 | C_32_H_50_O_7_ | -1.3 | 31.30 | +HCOO, -H | 367,2611; 191.0541 | Triterpenes | Alismatis rhizoma |
| 115 | Ginsenoside Rb3 | 1078.5887 | 1123.5869 | C_53_H_90_O_22_ | -3.2 | 31.36 | +HCOO, -H | 1077.5874; 783.4810; 621.4323; 459.3842 | Triterpenes | Ginseng radix et rhizoma |
| 116 | Malonyl Ginsenoside Rb2 | 1164.5875 | 1163.5803 | C_56_H_92_O_25_ | -4.5 | 31.51 | -H | 1077.5807; 945.5420; 783.4924; 621.4323 | Triterpenes | Ginseng radix et rhizoma |
| 117 | 20S-Quinquenoside R1 | 1150.6114 | 1195.6096 | C_56_H_94_O_24_ | -1.8 | 31.90 | +HCOO, -H | 1107.5935; 945.5420; 783.4810; 621.4426; 459.3842 | Triterpenes | Ginseng radix et rhizoma |
| 118 | Ophiogenin-3-O-α-L-Rhamnoside (1→2)-β-D-Rhamnoside | 754.4139 | 799.4105 | C_39_H_62_O_14_ | -1.1 | 32.02 | +HCOO | 445.2955; 143.0354; 113.0250 | [Steroids](https://www.medchemexpress.cn/NaturalProducts/NaturalProducts/steroids.html.html) | Ginseng radix et rhizoma |
| 119 | Ginsenoside Re | 946.5474 | 991.5456 | C_48_H_82_O_18_ | -2.7 | 32.18 | +HCOO, -H | 783.4924; 621.4374; 459.3798 | Triterpenes | Ginseng radix et rhizoma |
| 120 | Malonyl Ginsenoside Rd | 1032.5474 | 1031.5401 | C_51_H_84_O_21_ | -3 | 32.30 | -H | 945.5420; 927.5270; 783.4867 | Triterpenes | Ginseng radix et rhizoma |
| 121 | Ginsenoside Rs1 | 1120.6006 | 1165.5988 | C_55_H_92_O_23_ | -2 | 32.40 | +HCOO, -H | 1077.5740; 945.5420; 783.4924; 621.4374 | Triterpenes | Ginseng radix et rhizoma |
| 122 | 13β,17β-Epoxyalisol A | 506.3605 | 551.3587 | C_30_H_50_O_6_ | -0.4 | 32.92 | +HCOO, -H | 474.5568; 279.2312; 152.9966 | Triterpenes | Alismatis rhizoma |
| 123 | Ginsenoside Rg3 | 784.4946 | 829.4928 | C_42_H_72_O_13_ | -3.2 | 33.58 | +HCOO, -H | 783.4867; 621.4426; 459.3842; 375.2886 | Triterpenes | Ginseng radix et rhizoma |
| 124 | 20(R)Ginsenoside Rg3 | 784.4942 | 829.4927 | C_42_H_72_O_13_ | -3.4 | 33.65 | +HCOO, -H | 783.4867; 621.4323; | Triterpenes | Ginseng radix et rhizoma |
| 125 | 8-Formyl Ophiopogonanone B | 342.1103 | 341.1029 | C_19_H_18_O_6_ | -1.1 | 33.97 | -H | 325.0712; 208.0374; 177.0558 | Flavonoids; Phenols | Ophiopogonis radix |


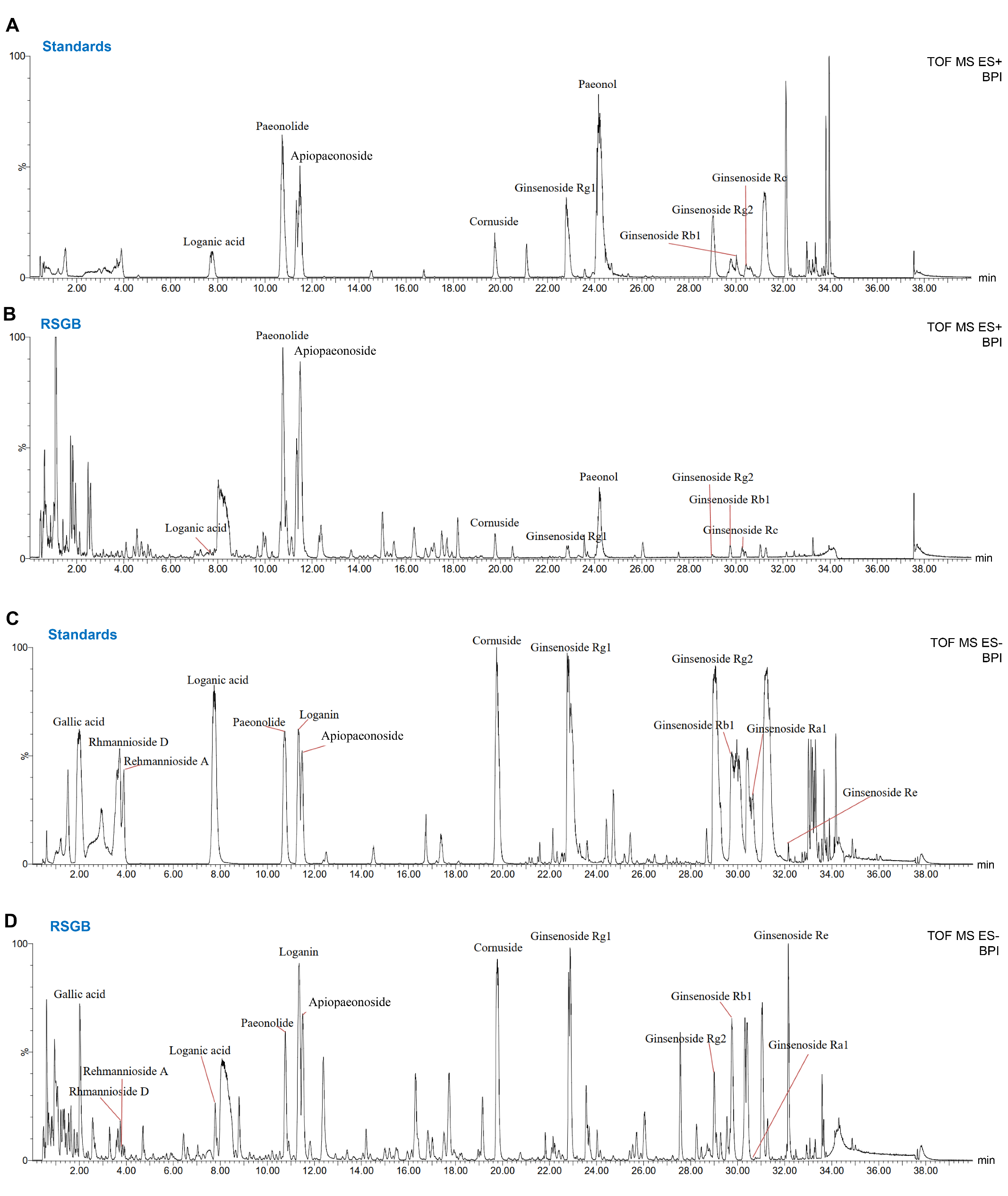


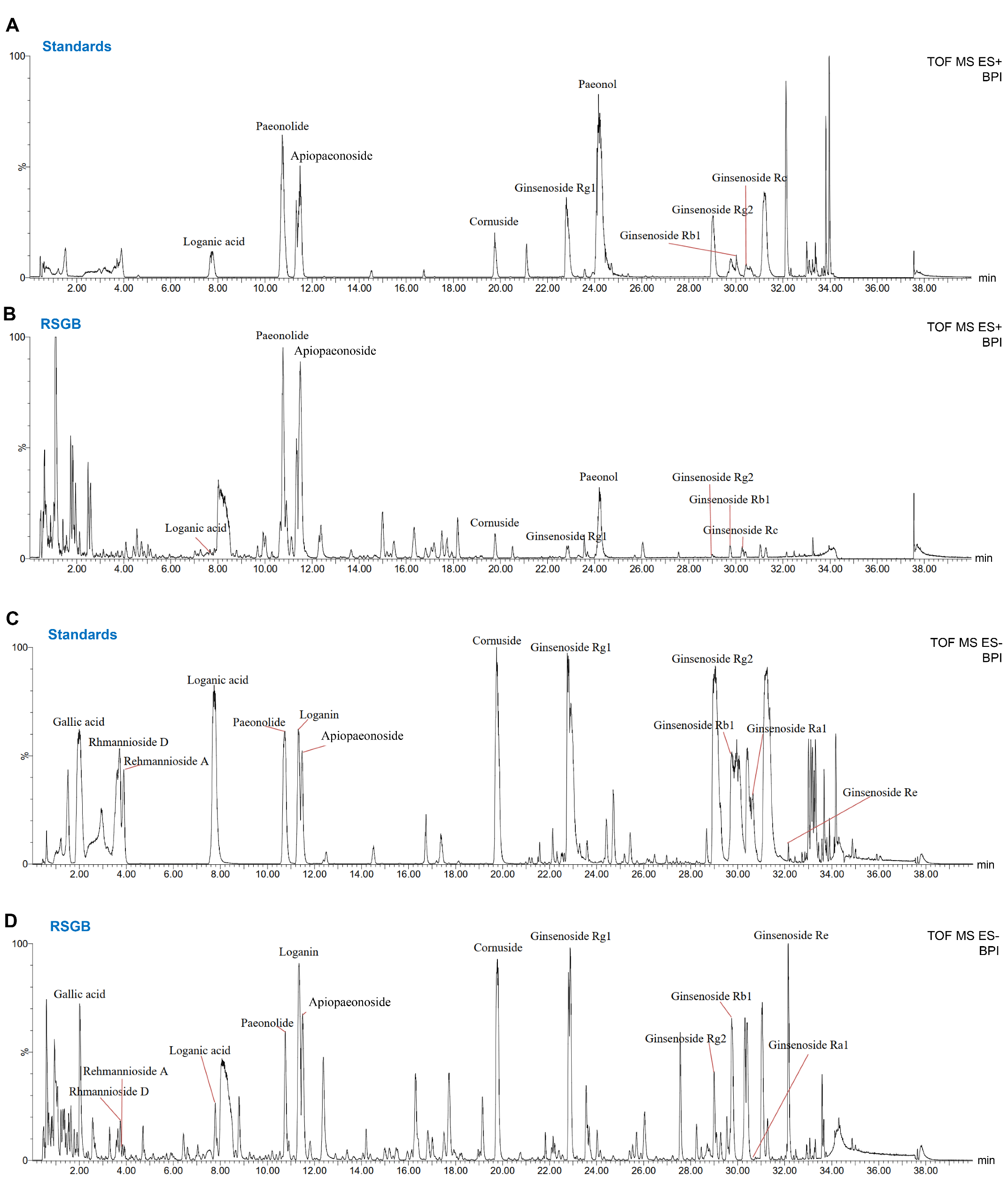


Fig S1. Fifteen standard mixtures and RSGB sample were detected by UPLC-QTOF-MS/MS. (A and B) BPI chromatograms of 15 standard mixtures and RSGB detected in positive ion mode, respectively. (C and D) BPI chromatograms of 15 standard mixtures and RSGB detected in negative ion mode, respectively.
